# DeePFAS: Deep-Learning-Enabled Rapid Annotation of PFAS: Enhancing Nontargeted Screening through Spectral Encoding and Latent Space Analysis

**DOI:** 10.1021/acs.est.5c09769

**Published:** 2025-09-30

**Authors:** Heng Wang, Tien-Chueh Kuo, Yufeng Jane Tseng

**Affiliations:** a Department of Computer Science and Information Engineering, 33561 National Taiwan University, Taipei 10617, Taiwan; b The Metabolomics Core Laboratory, Centers of Genomic and Precision Medicine, 33561 National Taiwan University, Taipei 10617, Taiwan; c Graduate Institute of Biomedical Electronics and Bioinformatics, 33561 National Taiwan University, Taipei 10617, Taiwan; d School of Pharmacy, College of Medicine, 33561 National Taiwan University, Taipei 10002, Taiwan

**Keywords:** PFAS (per- and polyfluoroalkyl substances), deep learning, mass spectrometry, environmental analysis, chemically latent space

## Abstract

Detecting PFAS is challenging due to their diverse chemical structures, lack of standards, complex sample matrices, and the need for sensitive equipment to measure trace levels. Background contamination and the sheer number of PFAS further hinder the development of a universal detection method. Liquid chromatography–high-resolution mass spectrometry (LC-HRMS) is the primary tool capable of analyzing PFAS in water, soil, and biological samples, and it is widely adopted in regulatory testing. However, LC-HRMS faces challenges, including contamination risk, labor-intensive preparation, low detection limits, and time-consuming data processing that requires advanced software and expertise to distinguish structurally similar compounds. To overcome these obstacles, we present DeePFAS, a deep-learning-based method for rapid annotation of PFAS. DeePFAS employs a spectral encoder integrating convolutional and transformer architectures to project raw MS2 spectra into a latent space of chemical structural features learned from a large corpus of unlabeled compounds. DeePFAS enables efficient annotation of MS2 spectra by comparing latent representations with candidate molecules, streamlining large-scale nontargeted PFAS screening, and reducing analytical complexity. Our method demonstrates the potential of AI-driven tools in environmental chemistry and is available at https://github.com/CMDM-Lab/DeePFAS.

## Graphical abstract

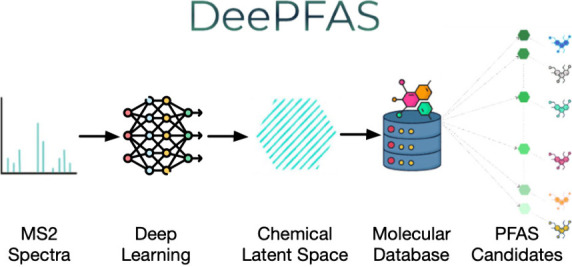

## Introduction

Detecting per- and polyfluoroalkyl substances (PFAS) and their transformation products (TP) in environmental and biological samples has emerged as a critical challenge in environmental analytical chemistry. Due to their unique physicochemical properties, PFAS represent a large class of synthetic chemicals widely employed in various products, such as waterproof and oil-resistant coatings and firefighting foams.^1–3^ Given the rarity of naturally occurring organofluorine compounds, detecting unknown organofluorine species in samples strongly implies a synthetic origin.^4–8^ Currently, the number of known PFAS compounds ranges from thousands to millions, depending on the definition and the source of information. According to the updated OECD definition, all chemicals containing a CF_3_ or isolated CF_2_ group are classified as PFAS, significantly increasing the total count of these compounds.^9,10^ The high stability of PFAS, attributed to the strong carbon–fluorine bonds, results in their persistence in the environment. This leads to their widespread detection in animals, soil, sediments, surface, ground, and drinking water. Such widespread detection raises significant public health concerns. These persistent chemicals have been linked to serious health issues, including endocrine disruption and an increased risk of cancers.^1^ While routine targeted screening typically focuses on fewer than 50 analytes, it may miss a significant portion of organofluorine compounds. Given the scarcity of analytical reference standards for these compounds, nontargeted screening (NTS) methods utilizing high-resolution mass spectrometry (HRMS) have become increasingly vital for effective PFAS identification and analysis.^1,4,11^ In contrast, nontargeted screening can help account for this substantial portion (e.g., using extractable organic fluorine analysis) present in environmental and human samples.

NTS is typically a time-intensive and partially manual process. A significant challenge stems from the complex data processing and subsequent annotation required, particularly when analyzing xenobiotics. This challenge is further highlighted by the need to reduce the data set from approximately 5000 detected compounds to 10–100 identified analytes or fewer,^1,12^ a process heavily reliant on efficient data handling and accurate compound annotation. Therefore, effective prioritization techniques are essential for distinguishing matrix components from target analytes. For instance, PFΔScreen utilizes accurate mass and isotope pattern analysis from full-scan MS1 data to infer elemental compositions and employs mass defect filtering techniques (such as MD/C-m/C) to remove irrelevant detected features.^4^ MS2 fragmentation is commonly used to obtain structural information and enhance confidence in identifying target analytes.^1,4^ However, compared to MS1 information acquired through full-scan, MS2 data, which is acquired through data-dependent acquisition (DDA) or data-independent acquisition (DIA), is often limited, particularly for low-abundance ions.^1^ Several primary approaches have been developed to utilize MS^2^ data for PFAS identification.^1,3^ These include the diagnostic fragment approach,^1,13,14^ utilizing predefined mass lists for preliminary detection; the fragment ion tagging approach,^13,14^ relying on characteristic fragment ions or neutral losses without the need for prior database knowledge; and the mass difference approach,^1,11,13,14^ based on repeating units (e.g., CF2) to facilitate screening. Each method has inherent limitations, such as labor-intensive processes, dependence on fragment formation, or reliance on precise mass calculations.

Recent advancements in structural elucidation have leveraged machine learning and deep learning techniques to address traditional limitations in compound identification. Two notable emerging methods are CSI-FingerID^15^ and MIST,^16^ each offering unique approaches to the challenge of molecular structure prediction. These methods may offer potential utility for the annotation of PFAS. CSI-FingerID utilizes support vector machines, a machine learning technique, to predict molecular fingerprints from fragmentation spectra. This method enables chemical class prediction and de novo structure generation by assigning chemical formulas to spectral peaks, constructing fragmentation trees, and employing multiple kernel functions to select the most plausible tree.

In contrast, MIST adopts a deep learning approach, decomposing input spectra into granular features and applying a transformer model to predict molecular fingerprints. Both approaches have shown promising competitive results in structural elucidation tasks. However, these methods share a common challenge: their accuracy heavily relies on the correct assignment of chemical formulas. We observed that the accuracy of chemical formula identification by CSI:FingerID for PFAS compounds was insufficient for nontargeted comprehensive analysis (refer to the SI section “Formula Identification of PFAS by CSI:FingerID” for further details). Furthermore, the recently competitive method for formula identification, MIST-CF,^17^ assigns chemical formulas via energy-based modeling and generates formula candidates through combinatorics before training neural networks. MIST-CF limits the number of rare elements such as halogens (i.e., F, Cl, Br, I), allowing each to appear at most once to narrow the search space for molecular formulas. Even with relaxed constraints on the number of fluorine atoms, the use of COMMON and RDBEs (ring double-bond equivalents)^18^ to further filter candidate formulas still presents issues of combinatorial explosion or exclusion of correct formulas (refer to the SI section “Formula Identification of PFAS by MIST-CF” for a detailed explanation). This dependency underscores the need for robust validation techniques to assess prediction reliability, even when overall performance is high. Despite this limitation, these tools represent significant progress in computational structural elucidation, offering new possibilities for rapid and accurate compound identification.

We propose DeePFAS, a deep learning-based approach for tentative PFAS annotations to address this need. The method projects raw MS2 data into the latent space of chemical structures for PFAS, facilitating the inference of structurally similar compounds by comparing spectra to multiple candidate molecules within this latent chemical space. DeePFAS can rapidly interpret individual MS2 spectra without requiring the correct molecular formula. In this study, DeePFAS demonstrates its ability to cluster structurally similar candidate molecules, with particularly strong performance in capturing the distinctive structural characteristics of PFAS. This approach allows efficient annotation of MS2 spectra in large-scale nontargeted PFAS screening, significantly reducing analytical complexity.

## Materials and Methods

### Workflow Overview

As depicted in Figure 1, our workflow is structured into two main stages: training and inference. In the training stage, we assembled an MS2 spectrum data set, which includes NIST20,^19^ NIST PFAS,^20^ and the PFAS standards mixture (std_150 ^21^) for training the DeePFAS model. The data sets underwent preprocessing and filtering before the training process. Each spectrum was assessed based on three validity criteria: 1) the precursor *m*/*z* must be less than 1,000; 2) the ionization mode must be negative, with an [M-H]- adduct ion; 3) the collision energy should range from 10 to 50 eV. Valid spectra were partitioned at the compound level, oversampled for data balancing, and further processed by incorporating loss features. Before training the DeePFAS model, structural features of the molecules were automatically extracted using an autoencoder from a molecule data set sourced from the PubChem^22^ and ZINC-20 ^23^ databases. These extracted features served as target labels to align the representation of the input MS2 spectrum with the corresponding molecular structure during the training process.

**Figure 1:**
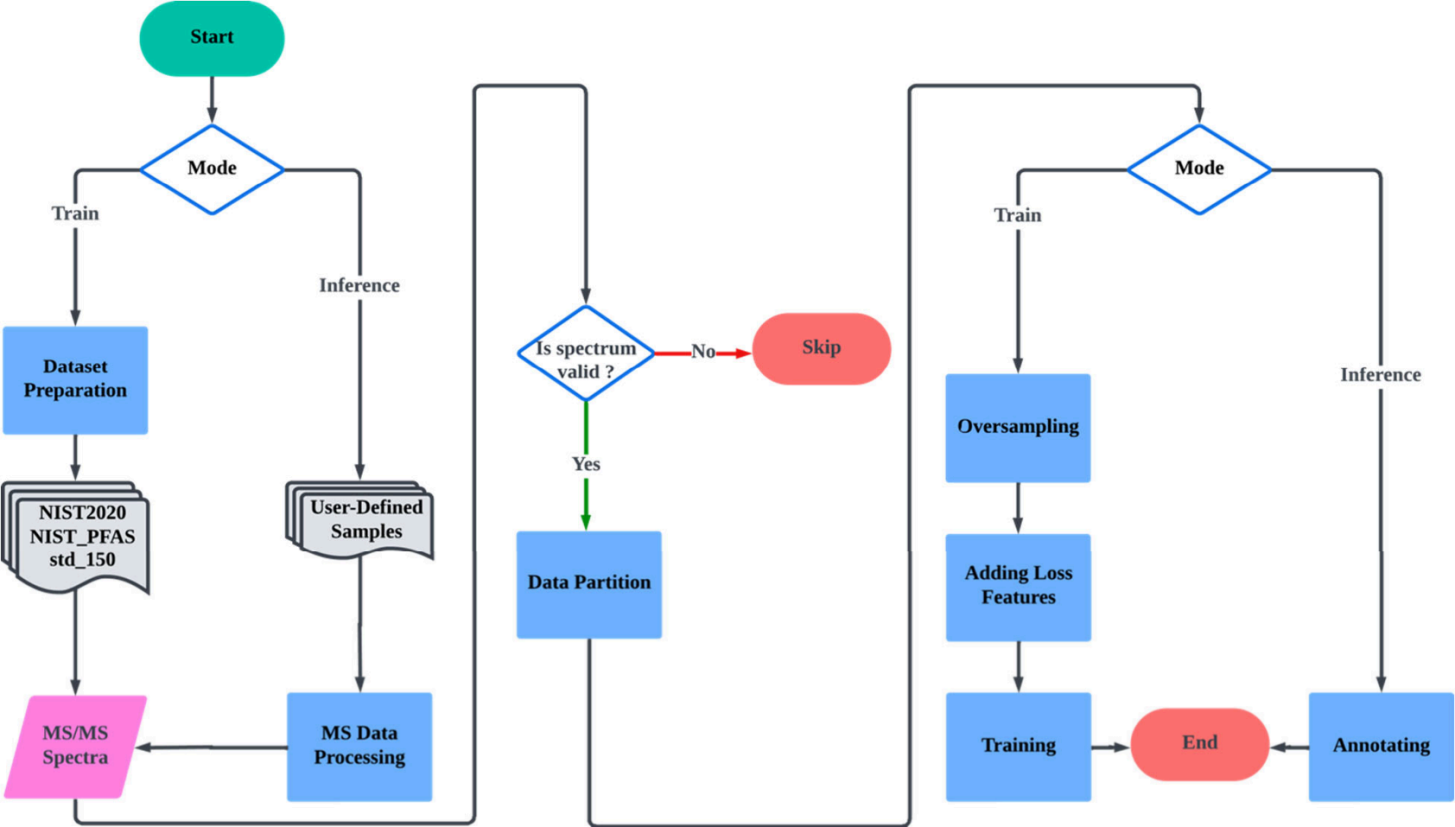
Overview of the workflow, comprising training and inference stages. In the training stage, high-quality MS2 spectra data sets are collected, filtered, and preprocessed for training the DeePFAS model. Valid spectra are partitioned, oversampled for data balancing, and processed by adding loss features. Before training, structural features of the molecules are extracted using an autoencoder from a molecule data set. These extracted features serve as target labels to align the representation of the input MS2 spectrum with the corresponding molecular structure. In the inference stage, the performance of DeePFAS is evaluated using user-defined samples.

In the inference stage, MS2 spectra from processed user-defined samples, obtained through MS Data Processing, are used to evaluate the performance of our model. Furthermore, all MS2 data were exported in MSP format and converted to MGF format for input into DeePFAS.

### MS Data Processing

In the inference stage, to assess the practical applicability of DeePFAS, we conducted a nontargeted analysis using an effluent sample (WWTP3) from industrial wastewater treatment plants (WWTPs), provided by Dr. Chen (Please ref to Supporting Information S6 and the doctoral dissertation of Dr. Chen^24^). The raw mass spectrometry data files of WWTP3 were converted to mzXML format using ProteoWizard (version 3).^25^ For targeted analysis of PFAS, precursor ion scans of all MS2 spectra were selected using the R (version 4.4.2) and R package XCMS (version 4.4.0).^26^ MS2 spectra were filtered based on a precursor *m*/*z* tolerance of 5 ppm relative to PFAS standards. The MS data analysis workflow comprised four steps: 1) raw data loading, 2) parameter optimization, 3) peak detection, and 4) spectra data export. (Please refer to the section ″ Supporting Information S7 “Parameter Settings for MS data processing” for details)

### Data Set Preparation for Training and Testing DeePFAS

For training and testing of our model, we assembled two distinct data sets: the Molecule Data set and the MS2 Spectrum Data set. The Molecule Data set comprised approximately 200 million molecules from the PubChem^22^ and ZINC-20 ^23^ databases, with each molecule represented using SMILES^27^ notation (Please refer to the section Supporting Information S9 Data Availability for details). Utilizing the RDKit toolkit (version 2023.9.5),^28^ we removed stereochemistry and salt information from the molecular representations and SMILES strings that RDKit could not parse. The removal of salt information was necessary to exclude disconnected SMILES representations (i.e., those containing a dot). In contrast, stereochemistry information was omitted due to the inherent challenges and infeasibility of recovering stereochemical configurations solely from mass spectrometry data. As a result, stereochemical analysis is beyond the scope of this study. Additionally, we focus exclusively on small molecules with molecular weights below 1000 Da, composed of common elements, including C, N, H, O, P, S, Cl, Br, F, I, and B. To ensure the stability of model training, we set the maximum allowed length for tokenized SMILES to 110 tokens, where each character or symbol—such as ‘C’, ‘c’, ‘Br’, ‘[’, and ‘)’—is treated as a single token. After filtering, the total number of molecules in the Molecule Data set did not change significantly, as most tokenized SMILES lengths were already below 100.

For the MS2 Spectrum Data set, we obtained spectral data from three distinct repositories. The NIST Tandem Mass Spectral Library 2020 (NIST20) provided over one million spectra from more than 30,000 compounds, serving as our primary commercial data set. Additionally, we incorporated data from the NIST PFAS database (Database Infrastructure for Mass Spectrometry - Per- and Polyfluoroalkyl Substances, National Institute of Standards and Technology), which is a publicly accessible and open-source HRMS database containing 24451 spectra from 132 PFAS compounds (accessed on January 2, 2025 for version 1.1). Furthermore, we included the PFAS standards mixture data set (std_150) from the National Environmental Research Academy, Ministry of Environment in Taiwan, which comprises 176 spectra from 27 PFAS compounds, specifically curated for PFAS annotation. This study focused exclusively on small molecules, excluding any spectra with large *m*/*z* values (greater than 1000 *m*/*z*). In our efforts to annotate PFAS, we selectively retained spectra obtained via the precursor ion in negative ion mode with hydrogen, reflecting typical ionization conditions for PFAS. Given the significant disparities in mass spectra at different collision energies,^29^ we focused only on spectra within a specific collision energy range to stabilize the training process. Since the spectra in both the std_150 and NIST PFAS data sets have collision energies not exceeding 50 eV, spectra with collision energies above 50 eV were excluded.

Additionally, spectra with collision energies below 10 eV were excluded due to the limited number of diagnostic fragment features observed at lower energies, which could compromise accurate structural characterization and destabilize the training process. Specifically, we only selected spectra with the highest confidence level 1a (Confirmed by reference standard) and 1b (Indistinguishable from reference standard) (see Table S3 for a detailed explanation) from the NIST PFAS database. Following filtration, the data sets contained 9,864 unique molecules with 68,619 spectra, 39 unique molecules with 2,025 spectra, and 26 unique molecules with 170 spectra from NIST20, NIST PFAS, and std_150, respectively.

### Data Set Partition

After filtering and screening in negative ion mode with hydrogen adduct, we extracted 9,864 compounds, encompassing 68,619 spectra, from the NIST20 database. These spectra represented various collision energies. The spectra data set was initially partitioned at the compound-level with a random split based on the first 14 characters of the InChIKey (a hash representing atomic connectivity while ignoring stereochemistry and charge, referred to as ‘InChIKey2D’), allocating 90% to training and 10% to testing. Subsequently, 10% of the training set was randomly selected, using the same InChIKey criteria, to form the validation set for model parameter tuning. As a result, the training, validation, and test sets comprised 7,990 (55,285 spectra), 888 (6,284 spectra), and 986 (7,050 spectra) unique molecules, respectively. To enhance the model’s capacity to annotate PFAS compounds, we incorporated molecules from the std_150 and NIST PFAS data sets into our training and test data sets. The std_150 and NIST PFAS data sets were also subjected to a random split, equally (50%/50%), using the same InChIKey-based method. After filtering, the std_150 data set contained 26 unique molecules (170 spectra), with 13 (55 spectra) included in the training set and the remaining 13 (115 spectra) in the test set. The NIST PFAS data set, after excluding compounds that overlapped with the std_150 data set, included 39 unique molecules (2,025 spectra). These were distributed with 20 (1,227 spectra) in the training set and 19 (798 spectra) in the test set. After these integrations, the final compositions of the training, validation, and test data sets were 8,023 (56,567 spectra), 888 (6,284 spectra), and 1,018 (7,963 spectra), respectively. We added no spectrum from the NIST PFAS or the std_150 data set to the validation set.

### Oversampling

The number of spectra associated with each compound varied considerably due to repeated measurements and differences in experimental parameters. To improve the stability of model training, we standardized the number of spectra per compound. We replicated and randomly sampled the available spectra for compounds with fewer spectra than a predefined threshold until the desired number was reached. Conversely, a random subset equal to the threshold was retained for compounds with more spectra than the threshold. Notably, some compounds in the NIST PFAS data set exhibited exceptionally high spectra, with several exceeding 100 (Table S4). To avoid increasing the computational cost per training epoch, we set the threshold at 50 spectra per compound. This value exceeds the maximum number of spectra observed for any compound in the NIST20 and std_150 data sets. It represents trade-offs that ensure consistent data representation across data sets while maintaining computational efficiency.

### Adding Loss Features

Due to fragmentation of MS2, only ionized fragments were detected, while neutral (uncharged) fragments did not appear in the MS2 spectrum. These neutral fragments were referred to as “loss features”. The *m*/*z* of each peak’s loss feature was determined by calculating the difference between the *m*/*z* of the precursor ion and that of each detected peak. Consequently, we utilized the relative intensity of the corresponding peak to represent the relative intensity of the neutral fragment, thereby generating a new set of features for analysis.

### Constructing DeePFAS To Predict SMILES from MS2 Spectra for PFAS Annotations

To address the scarcity of high-resolution MS2 spectra and the consequent lack of training data, we focused on capturing structural features closely related to target molecules rather than attempting to predict exact molecular structures. Using a large data set of unlabeled compounds, we employed an unsupervised approach to pretrain an autoencoder (AE) (Figure 2). The AE pretraining involved the SMILES representation to its canonical and enumerated forms by augmentation of random SMILES to improve model performance.^30–32^ Since each compound could correspond to various randomized SMILES, these were generated randomly during training. The autoencoder projects molecules into a reduced vector space, also known as chemical latent space or embeddings, representing molecular features. This setup allows us to approximate these embeddings using a spectral encoder based on convolutional neural networks (CNNs) and attention mechanisms. This phase aims to minimize the mean squared error between the molecular embeddings generated by the autoencoder and the spectral embeddings (Figure 2). This strategy has been applied in research ranging from speech-to-text translation to interpreting spectra into chemical structures.^33^ Specifically, DeePFAS seeks to reduce the distance between these two sets of embeddings and retrieves candidate molecules based on spectral embeddings to infer potential molecular structures.

**Figure 2:**
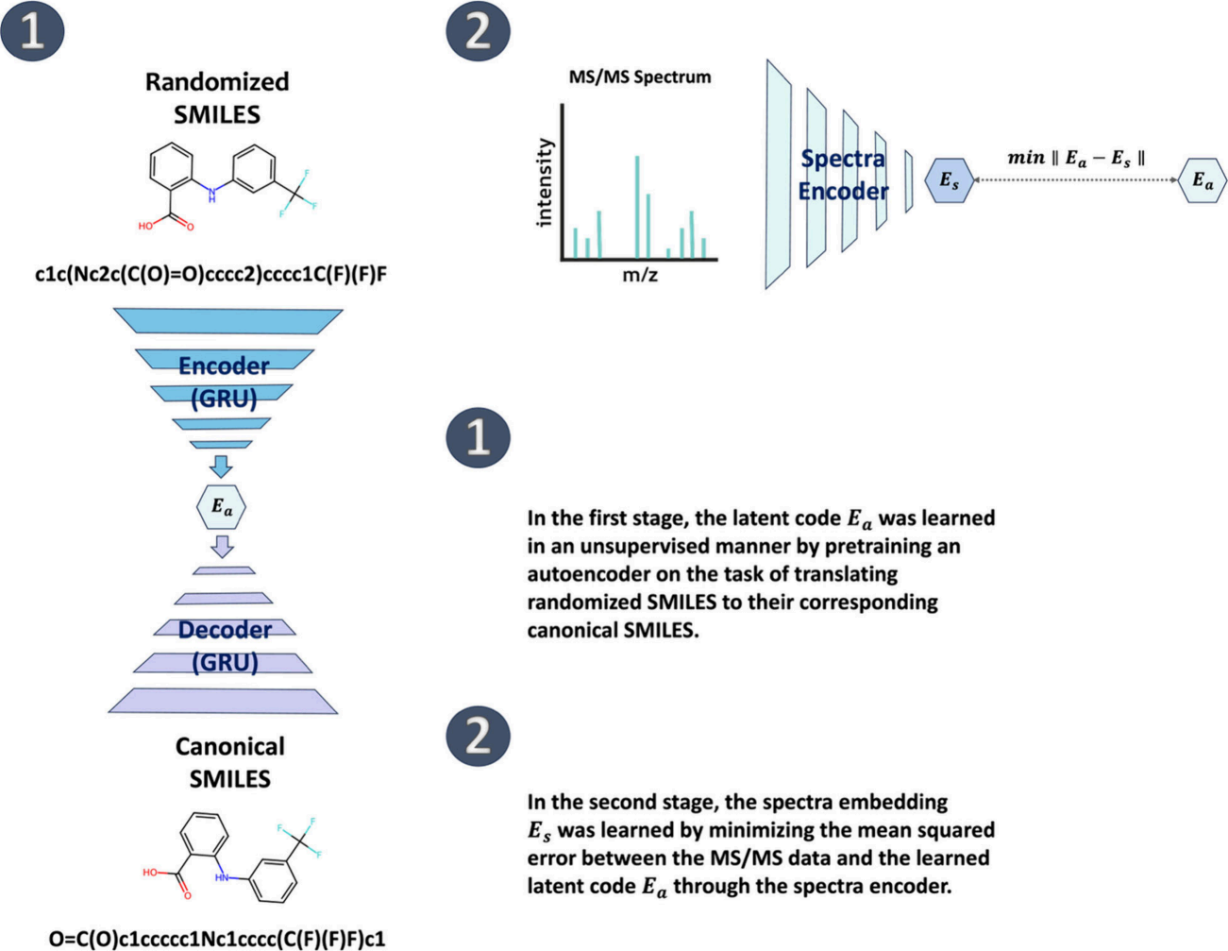
Conceptual overview of DeePFAS. We first performed unsupervised pretraining of an autoencoder by translating randomized SMILES into canonical SMILES to obtain the chemical latent code *E*_*a*_. Subsequently, the spectra embedding *E*_*s*_ was learned by minimizing the mean squared error between the MS2 data and the chemical latent code *E*_*a*_ through the spectra encoder.

### The Architecture of DeePFAS

DeePFAS comprises a pretrained autoencoder (AE) and an encoder that learns spectral embeddings. Both the encoder and decoder of the AE were based on gated recurrent units (GRUs), commonly used for learning sequential representations and tasks related to sequence-to-sequence models. GRUs were particularly effective due to their ability to manage the flow of information without the vanishing gradient problem often found in standard recurrent neural networks, making them suitable for tasks where understanding the context from long input sequences was crucial.^34,35^

The spectral encoder employed multiple attention layers combined with 2-D convolutional neural networks (CNNs) to achieve robust feature extraction from input spectra. Each peak in the spectra was represented as an (*m*/*z*, intensity) pair and was subsequently tokenized into a sequence based on the *m*/*z* value of the peak. Note that the value of *m*/*z* in each pair was rounded to two decimal places before being tokenized into a sequence, achieving a resolution of 0.01 Da. To effectively encode the relative intensity information between peak pairs within a spectrum, we implemented Rotary Positional Encoding (ROPE).^36^ ROPE modulated the query and key representations by rotating them at an angle proportional to the absolute position of each token in the input sequence, thereby establishing a relative positional encoding scheme that decayed with the increasing distance between tokens. This method allowed for dynamic adjustment of the encoding process, as the conventional fixed positional encoding coefficients typically used in ROPE were replaced by the normalized intensities of the input mass spectrum. The outputs from the attention layers were then channeled into 11 2-D CNN layers, each with different kernel sizes, to aggregate local information effectively. Since the runtime of DeePFAS primarily depends on the size of the molecular database, we evaluated its computational performance on a MacBook Air (M3) with 24GB of memory and a 512GB SSD. Under these conditions, the system can compute the mean squared error for approximately 690,000 chemical embeddings per second, based solely on CPU computation. More detailed information about the model architecture and training hyperparameters is provided in the Supporting Information section “Model Architecture and Optimization.”

### Definition of Metrics in Evaluations

The evaluation metrics were defined as follows:

- **Molecule Accuracy (MA)**: The percentage of unique compounds for which at least one of their measured spectra successfully identifies the correct molecule among the top 20 candidates.
- **Formula Accuracy (FA):** The percentage of unique compounds for which at least one of their measured spectra accurately identifies the correct molecular formula among the top 20 candidates.
- **Molecule Accuracy of Individual Spectra (MAS)**: The percentage of individual spectra accurately identifying the correct molecule among the top 20 candidates.
- **Formula Accuracy of Individual Spectra (FAS)**: The percentage of individual spectra accurately identifying the correct molecular formula within the top 20 candidates.
- **Confidence Level:** The number of spectra with all candidate molecules classified as PFAS, divided by the total number of spectra. The classification is based on the revised OECD definition for PFAS:^9^ “*PFASs are defined as fluorinated substances that contain at least a perfluorinated methyl group (−CF3) or a perfluorinated methylene group (−CF2−) (without any H/Cl/Br/I atom attached to the carbon within these groups)*”
- **Maximal Common Substructure (MCS)**: Employing the Spec2Mol metric,^33^ which involves calculating the MCS between two molecular structures using the RDKit Python package (version 2023.9.5). The substructure matches atom types, bond order, and ring structures, further assessed by three metrics: MCS ratio (***MCS*_*ratio*_**: $\frac{a_{MCS}}{a_{r}}$ ), MCS overlap coefficient (***MCS*_*ovrlap*_**: $\frac{a_{MCS}}{\min\left(a_{r},a_{p}\right)}$ ), and MCS Tanimoto (***MCS*_*tan*_**: $\frac{a_{MCS}}{a_{r}+a_{p}-a_{MCS}}$ ), where *a*_*MCS*_ represents the number of atoms in the MCS, *a*_*r*_ the number of atoms in the correct molecule, and *a*_*p*_ the number of atoms in the predicted molecule.
- **Fingerprint Similarity (FPS)**: Molecular fingerprints, which describe molecular structures as vector representations where each element signifies the presence of specific substructures or atomic/bond features, are widely used to determine molecular similarities. We assessed the average, maximum, and minimum similarities between the reference molecule and all candidates using the 2048-bit RDKit fingerprint available in the RDKit Python package.

## Results and Discussion

### Overview of DeePFAS

Following the proposed workflow, in the training mode, DeePFAS is optimized by minimizing the distance between SMILES embeddings generated by an Autoencoder (AE) and MS2 embeddings produced by the Spectra Encoder (SE). In inference mode, a trained DeePFAS model processes an MS2 spectrum as input by projecting it into a preconstructed latent space. The DeePFAS model identifies the 20 nearest chemical structures within this chemical latent space based on the shortest embedding distances by querying the PubChem reference database.

### Evaluation of DeePFAS Performance on the NIST20, NIST PFAS, and std_150 Data Sets

We evaluated the accuracy of molecular annotation under varying collision energies for the same molecule, maximal common substructure (MCS), and structural similarity of molecular fingerprints. Given the inherent limitations of MS2 spectra in completely reconstructing molecular structures, multiple molecular configurations could explain a single spectrum; thus, our analysis focused primarily on whether the model had learned to identify key structural features from the spectral data, rather than identifying structures identical to those in the data set.

To demonstrate the capabilities of DeePFAS, we independently evaluated its performance using the NIST20, NIST PFAS, and std_150 test data sets (Table 1). In the NIST20 test set, which comprised 986 unique molecules and 7,050 spectra, 34.7% of molecular identities and 66.4% of molecular formulas (MA/FA) were correctly identified within the top 20 candidate molecules. On the spectrum level, 23.5% of individual spectra correctly identified the molecule, and 50.7% identified the correct formula (MAS/FAS). For the std_150 test set, consisting of 13 unique molecules and 115 spectra, 61.5% of the molecular identities and formulas (MA/FA), and 39.1% of the spectra (MAS/FAS), were correctly identified. The NIST PFAS test set, which included 19 unique molecules and 798 spectra, 26.3% of molecular identities and formulas (MA/FA), and 10.4% and 11.1% of spectra (MAS/FAS), was correctly identified. To further evaluate the PFAS annotation capability of DeePFAS, we assessed the spectra of each compound individually within the std_150 and NIST PFAS test data sets. As shown in Table 2, compounds such as PFOS, PFHxS, PFDA, PFPeS, PFHpS, and PFNS had more than 50% of their spectra correctly identified within the top 20 candidate molecules. These PFAS belong to either the perfluoroalkyl sulfonic acids (PFSAs) or perfluoroalkyl carboxylic acids (PFCAs) classes, suggesting that DeePFAS effectively captures common structural features of typical PFAS compounds. However, the model faces challenges distinguishing more subtle structural variations—for example, the nonfluorinated three-carbon chains (CCC) in compounds like 4:2 FTSA and 8:2 FTSA. Regarding carbon chain length sensitivity, Figure 3 presents the top 20 candidate molecules retrieved for the measured spectrum of TFMS. It can be observed that all candidate molecules contain shorter perfluorinated carbon chains. This demonstrates that DeePFAS remains sensitive to variations in perfluorinated carbon chain lengths, even when the exact structure is not identified. Tables S4 and S5 indicate that the NIST PFAS test set exhibits more complex characteristics than the std_150 test set. Among these compounds, only Perfluorotetradecanoate and Perfluorotridecanoate, both classified as perfluoroalkyl carboxylic acids (PFCAs), displayed a higher proportion of spectra correctly identified (43.9% and 60.5%, respectively), consistent with the trends observed in the std_150 data set (Table S6). To further investigate the factors influencing performance, we conducted ablation experiments on (1) oversampling during training and (2) the mass resolution of spectra. DeePFAS was evaluated under three experimental configurations: (i) rounding the *m*/*z* to two decimal places and applying oversampling (named as R2-OS), (ii) rounding to two decimal places without oversampling (named as R2-NoOS), and (iii) rounding to one decimal place without oversampling (named as R1-NoOS). As shown in Table 1, the R2-OS configuration yielded optimal results for the NIST20 and std_150 data sets, whereas the R2-NoOS configuration performed better on the NIST PFAS data set. This discrepancy may stem from 36.8% of compounds in the NIST PFAS training set had more than 50 associated spectra, exceeding the threshold used in our oversampling strategy and resulting in partial data utilization (see Table S4). Achieving better data balance may require more sophisticated strategies, such as dynamically sampling spectra for each compound during training. In this study, however, we adopted a simpler oversampling approach for practical reasons. Structurally similar compounds may exhibit similar gradient behaviors during training, likely due to their shared fragmentation patterns, which could further complicate the effects of oversampling. As a result, oversampling in such contexts may not be straightforward. Exploring more advanced sampling strategies remains a promising direction for future research, particularly given the added complexity of training with spectra acquired under diverse experimental conditions. DeePFAS showed notably superior performance for spectral resolution with (R2-OS) and (R2-NoOS), suggesting that improved mass accuracy could offer richer features. However, due to hardware resource constraints, increasing resolution by one decimal place would exponentially increase token counts 10-fold; thus, higher-resolution experiments were not conducted. DeePFAS showed strong confidence in PFAS annotation across the std_150 and NIST PFAS data sets (Tables 2 and S6). In the std_150 data set, 69.2% of compounds had 100% annotation confidence, 84.6% exceeded 75%, and the lowest was 66.7%. Remarkably, all spectra in the NIST PFAS data set were annotated as PFAS compounds with 100% confidence. Additionally, all three test data sets exhibited high structural similarity, as evidenced by high Maximal Common Substructure (MCS) and Fingerprint Similarity (FPS) values displayed in Table 1, particularly in capturing core PFAS substructures, where higher values indicate stronger substructural and fingerprint-level similarities. Finally, we constructed a smaller reference database comprising approximately 50,000 non-PFAS compounds from PubChem, which was spiked with PFAS compounds from the std_150 and NIST PFAS data sets. The resulting PFAS annotation confidence levels were nearly identical to those obtained when searching the full PubChem database. This suggests that determining whether an MS2 spectrum corresponds to a PFAS compound may not require searching the entire PubChem database. This streamlined approach facilitates the rapid nontargeted screening of large-scale MS2 data sets.

**Table 1:** Performance Evaluation of DeePFAS on the NIST20, NIST PFAS, and std_150 Test Datasets under Different Experimental Configurations^a^

**Data set**	***MCS*_*ratio*_**	***MCS*_*tan*_**	***MCS*_*ovrlap*_**	***FPS*_*sim*_**	**MA (%)**	**FA(%)**	**FAS (%)**	**MAS (%)**	**Total number of molecules**	**Total number of MS** ^**2**^ **Spectra**
**NIST20 (R2-NoOS)**	Max: 0.76	Max: 0.66	Max: 0.79	Max: 0.63	34%	65.6%	49.9%	22.7%	986	7050
	Min: 0.46	Min: 0.33	Min: 0.51	Min: 0.27						
	Avg: 0.61	Avg: 0.47	Avg: 0.64	Avg: 0.43						
**NIST20 (R2-OS)**	**Max: 0.77**	**Max: 0.67**	**Max: 0.80**	**Max: 0.65**	**34.7%**	**66.4%**	**50.7%**	**23.5%**	986	7050
	**Min: 0.47**	**Min: 0.34**	**Min: 0.52**	**Min: 0.28**						
	**Avg: 0.62**	**Avg: 0.48**	**Avg: 0.65**	**Avg: 0.44**						
**NIST20 (R1-NoOS)**	Max: 0.75	Max: 0.65	Max: 0.78	Max: 0.61	31.6%	61.2%	43.6%	20.8%	986	7050
	Min: 0.45	Min: 0.32	Min: 0.50	Min: 0.26						
	Avg: 0.60	Avg: 0.45	Avg: 0.62	Avg: 0.41						
**NIST PFAS (R2-NoOS)**	Max: 0.86	Max: 0.74	**Max: 0.91**	**Max: 0.67**	**31.5%**	**36.8%**	**19.4%**	**17.5%**	19	798
	**Min: 0.62**	**Min: 0.46**	**Min: 0.72**	**Min: 0.32**						
	**Avg: 0.74**	**Avg: 0.59**	**Avg: 0.83**	**Avg: 0.49**						
**NIST PFAS (R2-OS)**	**Max: 0.87**	Max: 0.72	**Max: 0.91**	**Max: 0.67**	26.3%	26.3%	11.1%	10.4%	19	798
	Min: 0.57	Min: 0.40	Min: 0.63	Min: 0.29						
	**Avg: 0.74**	Avg: 0.57	Avg: 0.81	Avg: 0.47						
**NIST PFAS (R1-NoOS)**	Max: 0.86	**Max: 0.75**	**Max: 0.91**	Max: 0.65	26.3%	**36.8%**	11.9%	10.5%	19	798
	Min: 0.58	Min: 0.43	Min: 0.66	Min: 0.29						
	**Avg: 0.74**	**Avg: 0.59**	Avg: 0.80	Avg: 0.47						
**std_150 (R2-NoOS)**	Max: 0.83	Max: 0.74	Max: 0.87	Max: 0.70	61.5%	61.5%	37.3%	37.3%	13	115
	Min: 0.55	**Min: 0.40**	**Min: 0.62**	**Min: 0.26**						
	Avg: 0.70	**Avg: 0.55**	Avg: 0.75	**Avg: 0.50**						
**std_150 (R2-OS)**	**Max: 0.89**	**Max: 0.78**	**Max: 0.91**	**Max: 0.75**	61.5%	61.5%	**39.1%**	**39.1%**	13	115
	**Min: 0.62**	Min: 0.37	Min: 0.60	Min: 0.24						
	**Avg: 0.71**	**Avg: 0.55**	**Avg: 0.76**	**Avg: 0.50**						
**std_150 (R1-NoOS)**	Max: 0.80	Max: 0.73	Max: 0.85	Max: 0.69	**69.2%**	**69.2%**	31.3%	30.4%	13	115
	Min: 0.47	Min: 0.34	Min: 0.54	Min: 0.25						
	Avg: 0.69	Avg: 0.50	Avg: 0.69	Avg: 0.45						

a Metrics include Substructural Similarity, Chemical Fingerprint Similarity, and Annotation Accuracy. DeePFAS was assessed under three experimental settings: (1) rounding m/z of spectrum to two decimals with oversampling training dataset (R2-OS); (2) only rounding m/z of spectrum to two decimals (R2-NoOS); (3) only rounding m/z of spectrum to one decimal (R1-NoOS). The NIST20 and std_150 test datasets exhibited optimal performance with the DeePFAS under the experimental setting (R2-OS). Conversely, the NIST PFAS test dataset achieved its best results with DeePFAS under the experimental setting (R2-NoOS). The evaluation criteria for **Common Substructure** and **Chemical Fingerprint Similarity** are based on the average values calculated from all spectra associated with each compound, which are subsequently averaged across all compounds. **MCS**: maximal common substructure. **FPS**: fingerprint similarity. **MA**: the percentage of compounds for which at least one of their measured spectra successfully identifies the correct molecule among the top 20 candidates. **MAS**: the percentage of individual spectra that correctly identify the correct molecule among the top 20 candidates. **FA**: percentage of compounds for which at least one of their measured spectra accurately identifies the correct molecular formula among the top 20 candidates. **FAS**: percentage of individual spectra that accurately identify the correct molecular formula within the top 20 candidates. Cells highlighted in **bold** indicate the highest across different experimental settings.

**Table 2:** Evaluation of 13 PFAS standards in the Test Set Using Metric for Accessing Substructural Similarity, Chemical Fingerprint Similarity, and Annotation Accuracy^a^

**Compound**	***MCS*_*ratio*_**	***MCS*_*tan*_**	***MCS*_*ovrlap*_**	***FPS*_*sim*_**	**Number of MS2 spectra with correct identification**	**Confidence Level**	**Total number of MS2 spectra**	**Annotation Accuracy**
**TFMS**	Max: 0.93	Max: 0.48	Max: 0.93	Max: 0.23	0	100%	17	0%
	Min: 0.49	Min: 0.22	Min: 0.49	Min: 0.04				
	Avg: 0.72	Avg: 0.34	Avg: 0.72	Avg: 0.10				
**PFOS**	Max: 0.99	Max: 0.99	Max: 1.00	Max: 1.00	12	100%	13	92.3%
	Min: 0.67	Min: 0.62	Min: 0.89	Min: 0.37				
	Avg: 0.81	Avg: 0.76	Avg: 0.93	Avg: 0.77				
**N-EtFOSAA**	Max: 0.88	Max: 0.87	Max: 0.99	Max: 0.78	0	100%	3	0%
	Min: 0.60	Min: 0.54	Min: 0.85	Min: 0.28				
	Avg: 0.71	Avg: 0.66	Avg: 0.89	Avg: 0.47				
**PFPrA**	Max: 0.86	Max: 0.63	Max: 0.86	Max: 0.54	3	100%	9	33.3%
	Min: 0.60	Min: 0.31	Min: 0.60	Min: 0.11				
	Avg: 0.74	Avg: 0.43	Avg: 0.74	Avg: 0.23				
**PFHxS**	Max: 0.98	Max: 0.96	Max: 0.99	Max: 0.95	10	100%	12	83.3%
	Min: 0.60	Min: 0.46	Min: 0.66	Min: 0.32				
	Avg: 0.81	Avg: 0.69	Avg: 0.85	Avg: 0.71				
**PFDA**	Max: 0.77	Max: 0.74	Max: 0.80	Max: 0.80	2	75%	4	50%
	Min: 0.44	Min: 0.35	Min: 0.53	Min: 0.28				
	Avg: 0.59	Avg: 0.53	Avg: 0.70	Avg: 0.58				
**FBSA**	Max: 0.95	Max: 0.73	Max: 0.95	Max: 0.82	5	100%	20	25%
	Min: 0.68	Min: 0.41	Min: 0.68	Min: 0.26				
	Avg: 0.82	Avg: 0.54	Avg: 0.82	Avg: 0.47				
**PFPeS**	Max: 1.00	Max: 1.00	Max: 1.00	Max: 1.00	5	100%	5	100%
	Min: 0.64	Min: 0.44	Min: 0.64	Min: 0.27				
	Avg: 0.85	Avg: 0.69	Avg: 0.85	Avg: 0.64				
**PFHpS**	Max: 1.00	Max: 1.00	Max: 1.00	Max: 1.00	5	100%	5	100%
	Min: 0.53	Min: 0.36	Min: 0.56	Min: 0.29				
	Avg: 0.81	Avg: 0.70	Avg: 0.85	Avg: 0.67				
**PFNS**	Max: 0.88	Max: 0.94	Max: 0.99	Max: 1.00	3	100%	5	60%
	Min: 0.56	Min: 0.43	Min: 0.66	Min: 0.35				
	Avg: 0.74	Avg: 0.70	Avg: 0.91	Avg: 0.79				
**FBSE**	Max: 0.95	Max: 0.66	Max: 0.83	Max: 0.66	0	81.2%	16	0%
	Min: 0.55	Min: 0.27	Min: 0.43	Min: 0.22				
	Avg: 0.76	Avg: 0.44	Avg: 0.62	Avg: 039				
**4:2 FTSA**	Max: 0.79	Max: 0.67	Max: 0.80	Max: 0.51	0	66.7%	3	0%
	Min: 0.35	Min: 0.21	Min: 0.35	Min: 0.16				
	Avg: 0.52	Avg: 0.36	Avg: 0.52	Avg: 0.28				
**8:2 FTSA**	Max: 0.65	Max: 0.53	Max: 0.68	Max: 0.46	0	66.7%	3	0%
	Min: 0.27	Min: 0.19	Min: 0.35	Min: 0.20				
	Avg: 0.47	Avg: 0.36	Avg: 0.54	Avg: 0.34				

a PFOS, PFHxS, PFDA, PFPeS, PFHpS, and PFNS had more than 50% of their spectra correctly identified within the top20 candidate molecules. 69.2% of compounds had 100% annotation confidence, 84.6% had annotation confidence exceeding 75%, and the lowest confidence level was 66.7%. The evaluation criteria for **Common Substructure** and **Chemical Fingerprint Similarity** are based on the average values obtained from all spectra for each compound. **MCS**: maximal common substructure. **FPS**: fingerprint similarity. **Confidence Level**: Number of spectra with all candidates classified as PFAS, divided by the total number of spectra. **Annotation Accuracy**: number of MS^2^ spectra with correct identification, divided by the total number of spectra.

**Figure 3:**
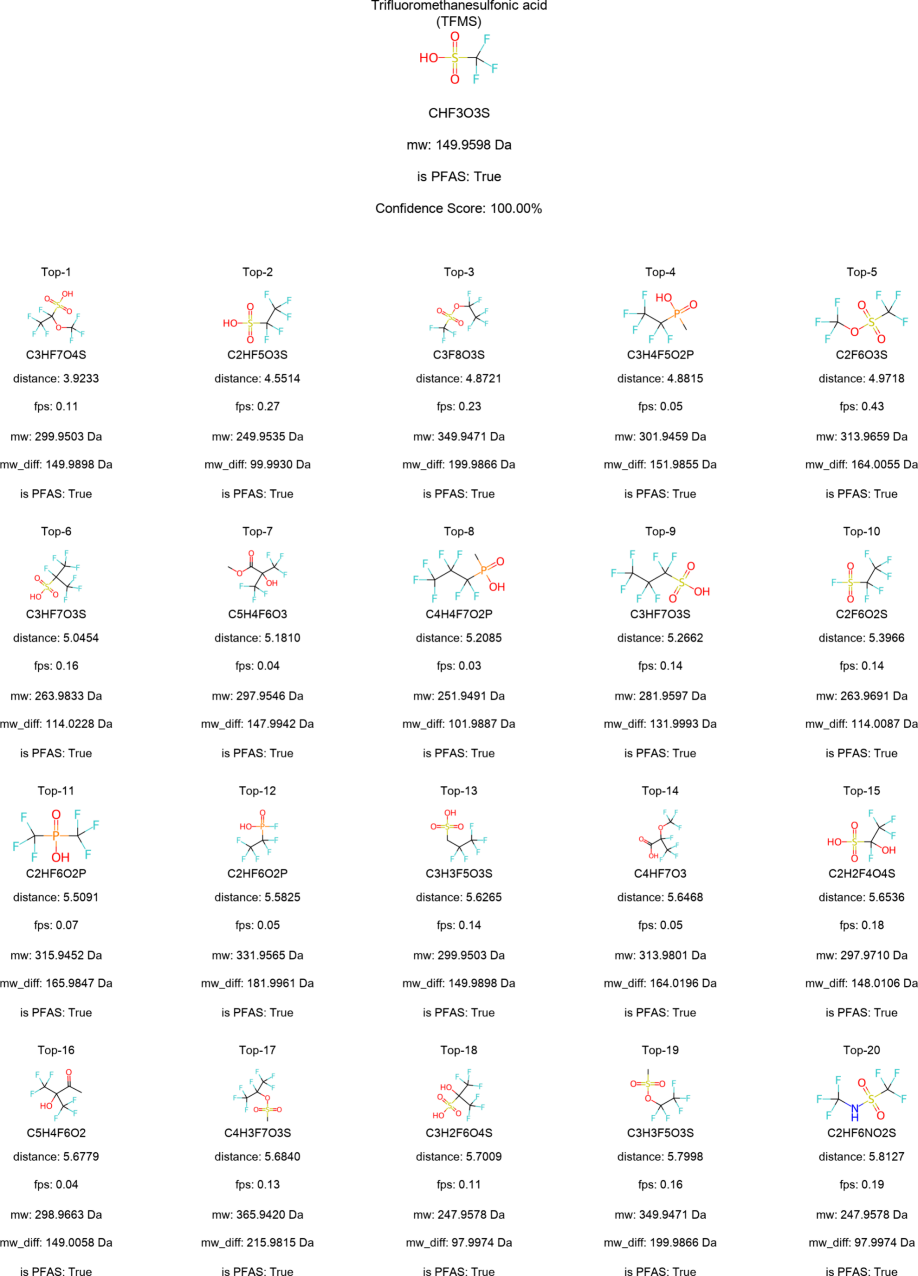
Twenty candidate molecules were generated by DeePFAS from an input of one measured spectrum of TFMS. The candidates are ranked based on their embedding distance (mean squared error) to the correct molecule, with the shortest distance corresponding to the highest rank. Each column displays the molecular formula, molecular weight (**mw**), embedding distance (**distance**), fingerprint similarity (**fps**), classification as PFAS (**is PFAS**) according to the revised OECD definition for PFAS, and the mass difference (**mw_diff**) relative to the correct molecule. The **Confidence Score** is calculated as the percentage of candidate molecules classified as PFAS (**is PFAS**) relative to the total number of candidates.

### Evaluation with a Wastewater Sample

To assess the practical applicability of DeePFAS, we conducted a nontargeted analysis of a wastewater sample (WWTP3) provided by Dr. Chen (Please ref to Supporting Information S6 and the doctoral dissertation of Dr. Chen^24^). The sample comprised 12,053 MS2 spectra acquired through peak detection using optimized parameters. According to the work conducted by Chen et al., a total of 83 PFAS compounds, representing 12 subclasses across 3 primary classes, were identified with varying levels of confidence through targeted and nontargeted analyses in industrial sewage and effluents from associated wastewater treatment plants (WWTPs). Identified PFAS matched with available authentic standards were categorized as CL1a/CL1b (denoted as “CL1-identified PFAS”), while those lacking authentic standard confirmation were classified as CL2b to CL3c (denoted as “tentatively identified PFAS”). Among the 12,053 MS2 spectra, 648 corresponded to 15 CL1-identified PFAS, and 86 spectra matched 24 tentatively identified PFAS (Tables S7 and S9). For the 15 CL1-identified PFAS, compounds such as 6:2 FTSA, PFHpA, PFHxA, PFBS, PFPeA, PFBSi, and PFBA had over 50% of their spectra correctly identified by DeePFAS among the top 20 candidate molecules. 86.7% of these compounds showed annotation confidence exceeding 90%, and 93.3% exceeded 50% confidence (Table S8). For the 24 tentatively identified PFAS compounds, 79.1% achieved 100% annotation confidence, and 95.8% exhibited confidence levels exceeding 50%. However, no PFAS compound was correctly identified among the candidate molecules, suggesting that while DeePFAS demonstrates sensitivity in detecting PFAS features, accurately determining molecular structures remains challenging. To further evaluate DeePFAS, we compared its performance with PFΔScreen^4^—a Python-based, open-source tool featuring a user-friendly GUI for prioritizing features through several PFAS-specific analytical strategies. These include MD/C-m/C filtering, Kendrick mass defect (KMD) analysis, diagnostic fragment identification, fragment mass difference matching, and suspect screening. PFΔScreen operates in two phases: *FeatureFinding* and *PFASPrioritization*. The first phase uses the FeatureFinderMetabo algorithm on MS1 data to detect features, after which MS2 spectra are aligned to precursors using *m*/*z* and RT tolerances. Multiple evidence metrics for MS1 features—such as carbon count, MD, MD/C, m/C, KMD, and homologous series (HS)—are calculated in the second phase. For MS2 spectra, it matches fragment mass differences against predefined PFAS-specific patterns and identifies diagnostic fragments (DFs). Only the single MS2 spectrum with the highest precursor intensity is retained per MS1 feature. For a fair comparison, PFΔScreen annotated a PFAS if its theoretical *m*/*z* matched within a 5 ppm tolerance, and either a diagnostic fragment or fragment mass difference was detected, or it matched a suspect PFAS entry. DeePFAS, in contrast, required a ≥ 90% annotation confidence for an identification. Among the 15 CL1-identified PFAS, DeePFAS successfully annotated 13 compounds, while PFΔScreen identified all 15. As shown in Table S8, the PFAS annotation confidence levels produced by DeePFAS for PFPrA and PFHxA did not exceed 50%. This may be attributed to matrix effects or low concentrations, which can enhance or suppress certain fragment ion signals, ultimately affecting the model’s prediction accuracy (Please refer to Supporting Information S6). Figures S1–S4 and S5–S6 illustrate comparisons between four MS2 spectra of PFPrA and two MS2 spectra of PFHxA obtained from WWTP3 and those from authentic standards. Several peaks not present in the reference standard spectra were markedly intensified among the top four WWTP3 samples. Conversely, suppression of certain fragment ions was also observed—for instance, the fragment ion at *m*/*z* 118.9925 in one of the WWTP3 spectra was notably suppressed (Figure S4). These prediction results appear consistent with expectations, as matrix effects and low concentrations are known to impact fragment ion intensities, thereby reasonably explaining the observed reduction in annotation confidence. Interestingly, the precursor ion of FBSEE diol appeared as an acetate adduct [M+CH_3_COO]^−^, differing from the assumed [M – H]^−^. Both DeePFAS and PFΔScreen still annotated this as PFAS, suggesting that MS2 spectra with different adducts may retain sufficient PFAS-specific features for annotation, indicating future potential for DeePFAS to generalize across precursor ion types. Among the 24 tentatively identified PFAS compounds, DeePFAS successfully annotated 19 as PFAS, whereas PFΔScreen identified 13 as PFAS (Table S10). The lower number of annotations by PFΔScreen can be attributed to the absence of characteristic diagnostic fragments and fragment mass differences. Specifically, five compounds from the 24 tentatively identified PFAS compounds were excluded due to the lack of these typical fragment patterns. These two types of evidence are widely regarded as important indicators for PFAS annotation. However, expanding existing libraries of diagnostic fragments presents inherent limitations. Therefore, tools like DeePFAS—which rely solely on MS2 spectral information—offer an attractive alternative for PFAS annotation. To explore unidentified PFAS, we clustered unmatched MS2 spectra based on their precursor *m*/*z* values. Specifically, two spectra with precursor *m*/*z* values denoted as *pm*_*1*_ and *pm*_*2*_ were grouped into the same cluster if their mass difference satisfied the criterion: *abs­(pm*_*1*_ *–pm*_*2*_ *)/max­(pm*_*1*_ *, pm*_*2*_ *) × 10* ^*6*^ *≤ 5 ppm*. Spectra were grouped under the assumption that clusters represent either the same compound or structurally similar isomers. Relaxed inclusion criteria were applied: for DeePFAS, only spectra with annotation confidence ≥80% were considered, while PFΔScreen required the presence of an MS1–MS2 match. Across the entire wastewater sample, a total of 4,486 clusters were formed. After excluding 39 associated with previously identified PFAS, 4,447 clusters remained for comparison. DeePFAS and PFΔScreen annotated 949 and 987 clusters as PFAS-related, respectively. Notably, 62.3% (591 clusters) of the PFAS-annotated clusters by DeePFAS overlapped with those annotated by PFΔScreen. This overlap suggests the potential presence of additional PFAS derivatives in the sample. However, the nonoverlapping annotations point to possible inconsistencies and emphasize the need to address false positives. These findings highlight that while DeePFAS demonstrates utility in nontargeted PFAS annotation, it still faces limitations and uncertainties that require further refinement.

### DeePFAS as a Viable Solution for Addressing the Complexities of Nontargeted PFAS Screening

This study proposes a novel deep learning-based approach, DeePFAS, which projects MS2 data into a latent chemical structure space. By comparing spectra within this latent space, the proposed method enables the inference of structurally similar compounds, thus enhancing the accuracy and confidence of PFAS identification. Furthermore, DeePFAS demonstrated sensitivity in detecting PFAS-specific features and practical feasibility through its application to wastewater samples. This approach allows efficient annotation of MS2 spectra in large-scale nontargeted PFAS screening, significantly reducing analytical complexity. However, as previously mentioned, certain limitations remain, particularly related to false-positive annotations. As shown in Table 2, a few PFAS compounds in the standard test set were annotated by DeePFAS with relatively low confidence levels. We hypothesize that the low sensitivity of PFAS annotation may contribute to the increased false positive rate, as it indicates that DeePFAS may not effectively capture the characteristic features of certain PFAS compounds. Expanding the existing library of known spectra through in silico methods and utilizing unannotated MS2 spectra for model pretraining may offer feasible solutions to address these limitations.^37–40^ Furthermore, considering the notable discrepancies between DeePFAS and existing tools in nontargeted annotation, comparing annotation results from multiple tools could also prove beneficial. This approach, designed to enhance nontargeted PFAS analysis, is available for further research and development at https://github.com/CMDM-Lab/DeePFAS.

## Supplementary Material



## Notes

Detailed descriptions of the model architecture, training hyperparameters, chemical lists, code and data availability, performance evaluation tables, and comparative figures of MS2 spectra (PDF)
