# Supplementary material for: DeePFAS: Deep-Learning-Enabled Rapid Annotation of PFAS: Enhancing Nontargeted Screening through Spectral Encoding and Latent Space Analysis
Source: Environ Sci Technol. 2025 Sep 30;59(46):24841–52. doi: 10.1021/acs.est.5c09769 (PMC12659435; doi:10.1021/acs.est.5c09769)
Supplement: Supplementary file 1 [file es5c09769_si_001.pdf]

Supporting Information: DeePFAS: Deep Learning-Enabled Rapid Annotation of PFAS: Enhancing Non-Targeted Screening through Spectral Encoding and Latent Space Analysis

Heng Wang[a], Tien-Chueh Kuo[b], Yufeng Jane Tseng[, a, b, c, d]*

[a]Department of Computer Science and Information Engineering, National Taiwan University, Taipei 10617, Taiwan

[b]The Metabolomics Core Laboratory, Centers of Genomic and Precision Medicine, National Taiwan University, Taipei 10617, Taiwan

[c]Graduate Institute of Biomedical Electronics and Bioinformatics, National Taiwan University, Taipei 10617, Taiwan

[d]School of Pharmacy, College of Medicine, National Taiwan University, Taipei 10002, Taiwan

[*]Corresponding Author

Voice: +886.2.3366.4888#529

Fax: +886.2.23628167

E-mail: yjtseng@csie.ntu.edu.tw

Contents

S1 Formula Identification of PFAS by CSI:FingerID S4
S2 Formula Identification of PFAS by MIST-CF S5
S3 Parameter Settings for MS data processing.......................................S7
S4 HRMS Data from Standards Dataset std_150...................................S8
S5 HRMS Data from NIST PFAS Database S13
S6 HRMS Data from a Wastewater Sample (WWTP3).......................S20
S7 Parameter Settings for PFΔScreen in the Comparative Analysis with DeePFAS S35
S8 Model Architecture and Optimization..S36
S9 Data Availability S38
S10 Code Availability S39
S11 Supplementary References...S40

Number of Figures: 7
Number of Tables: 11
Number of Pages: 41

List of Tables

Table S1: Chemicals list of the training set of the std_150 dataset. It includes 13 unique PFAS compounds with chemical name, molecular formula, molecular weight, canonical SMILES, PubChem CID, and the number of measured spectra (a total of 55 spectra) S9
Table S2: Chemicals list of the testing set of the std_150 dataset. It includes 13 unique PFAS compounds with chemical name, molecular formula, molecular weight, canonical SMILES, PubChem CID, and the number of measured spectra (total 115 spectra) S11
Table S3: Criteria for PFAS Identification at Various Confidence Levels and Statistical Number of Spectra in NIST PFAS Database...S13
Table S4:: The chemical list of the training set from the NIST PFAS dataset. It includes 20 unique PFAS compounds with chemical name, molecular formula, molecular weight, canonical SMILES, PubChem CID, and the number of measured spectra (a total of 1227 spectra) S14
Table S5: Chemical list of the testing set from the NIST PFAS dataset. It includes 19 unique PFAS compounds with chemical name, molecular formula, molecular weight, canonical SMILES, PubChem CID, and the number of measured spectra (total 798 spectra) S16
Table S6: Evaluation of 19 PFAS compounds in the Test Set of NIST PFAS Database Using Metric for Accessing Substructural Similarity, Chemical Fingerprint Similarity, and Annotation Accuracy S18
Table S7: List of identified PFAS compounds categorized as CL1a/CL1b based on matching with available authentic standards in wastewater samples from WWTP3. The table includes 15 unique PFAS compounds, their chemical names, molecular formulas, molecular weights, canonical SMILES representations, and the number of measured spectra (a total of 648).S21
Table S8: Evaluation of 15 identified PFAS compounds categorized as CL1a/CL1b based on matching with available authentic standards in wastewater samples from WWTP3. The evaluation was performed using metrics for assessing substructural similarity, chemical fingerprint similarity, and annotation accuracy. Compounds such as 6:2 FTSA, PFHpA, PFHxA, PFBS, PFPeA, PFBSi, and PFBA had more than 50% of their spectra correctly identified within the top 20 candidate molecules. 86.7% of the compounds showed annotation confidence greater than 90%, while 93.3% exhibited confidence exceeding 50%. S23
Table S9: List of identified PFAS compounds categorized as CL2b to CL3c in wastewater samples from WWTP3. This table includes 24 unique PFAS compounds, their chemical names, molecular formulas, molecular weights, canonical SMILES representations, and the number of measured spectra (a total of 86 spectra). For detailed information, please refer to Table 24 and the appendix section in the doctoral dissertation of Dr. Chen (https://tdr.lib.ntu.edu.tw/retrieve/e6a0d00b-326e-41e4-bc87-1567f316c5e5/ntu-112-2.pdf). S28
Table S10: Evaluation of 24 identified PFAS compounds categorized as CL2b to CL3c in wastewater samples from WWTP3. The evaluation was performed using metrics for assessing substructural similarity, chemical fingerprint similarity, and annotation accuracy. 79.1% of the compounds had 100% annotation confidence, while 95.8% exhibited confidence exceeding 50%. 13 PFAS compounds were identified by PFΔScreen. S33
Table S11: Model hyperparameters searched for the autoencoder and spectra encoder............S37

List of Figures

Figure S1: Comparison of MS/MS Spectra of PFPrA from std_150 and WWTP3 Samples. Peak intensities were normalized to the maximum intensity within each spectrum. The four most intense peaks were annotated with their corresponding m/z values. S25
Figure S2: Comparison of MS/MS Spectra of PFPrA from std_150 and WWTP3 Samples. Peak intensities were normalized to the maximum intensity within each spectrum. Additionally, Theense peaks were annotated with their corresponding m/z values. S26
Figure S3: Comparison of MS/MS Spectra of PFPrA from std_150 and WWTP3 Samples. Peak intensities were normalized to the maximum intensity within each spectrum. The four most intense peaks were annotated with their corresponding m/z values. S26
Figure S4: Comparison of MS/MS Spectra of PFPrA from std_150 and WWTP3 Samples. Peak intensities were normalized to the maximum intensity within each spectrum. Additionally, Theense peaks were annotated with their corresponding m/z values. S27
Figure S5: Comparison of MS/MS Spectra of PFHxA from std_150 and WWTP3 Samples. Peak intensities were normalized to the maximum intensity within each spectrum. The four most intense peaks were annotated with their corresponding m/z values. S28
Figure S6: Comparison of MS/MS Spectra of PFHxA from std_150 and WWTP3 Samples. Peak intensities were normalized to the maximum intensity within each spectrum. The four most intense peaks were annotated with their corresponding m/z values. S28
Figure S7: The Parameter Settings for PFΔScreen in the Comparative Analysis with DeePFAS. S35

S1 Formula Identification of PFAS by CSI:FingerID

To assess the ability of CSI:FingerID in formula identification for PFAS, we selected the filtered std_150 and NIST PFAS datasets mentioned in the Dataset Partition section of the original text. We used SIRIUS version 6.1.0 (which integrates CSI:FingerID) to predict the chemical formulas for the std_150 and NIST PFAS datasets. The prediction was performed under the conditions allowing the atomic types C, H, O, N, P, I, B, Br, Cl, F, and S (with no quantity restrictions) and setting the MS2 mass accuracy to ≤ 10 ppm. The formula prediction accuracy for the filtered std_150 and NIST PFAS datasets was 68.8% and 31.25%, respectively. Additionally, we evaluated the proportion of predicted formulas that contained fluorine. The results showed that 75.3% of spectra from the std_150 dataset and 67.45% from the NIST PFAS dataset included fluorine in their predicted molecular formulas.

S2 Formula Identification of PFAS by MIST-CF

Since MIST-CF assigns chemical formulas through energy-based modeling, the method requires generating formula candidates via combinatorial approaches before training the neural networks. The services provided by SIRIUS facilitate the generation of molecular formula candidates in MIST-CF. To narrow the search space for molecular formulas, MIST-CF imposes constraints on the number of rare elements, such as halogens (e.g., F, Cl, Br, I), limiting them to appear at most once. Additionally, filtering methods, such as the COMMON and RDBE (Ring Double-Bond Equivalents) filters in SIRIUS, reduce the number of candidate formulas by excluding chemically infeasible molecular formulas. However, PFAS compounds often contain large amounts of fluorine, and we found that, even after filtering, the number of candidate formulas remains excessively high. For example, we tested PFDoA (Perfluoro-n-dodecanoic acid) using SIRIUS (version 6.1.0) on one of its spectral measurements. We used the same configuration as in MIST-CF, except for the number of fluorine atoms. No restrictions were applied to the number of common elements such as C, N, O, or H. The maximum allowed number of sulfur (S) and phosphorus (P) atoms was set to 5 and 3, respectively. At the same time, each halogen, except for fluorine (i.e., Cl, Br, I), was limited to one per formula. The maximum number of fluorine (F) atoms allowed was 25 for PFAS. After selecting candidates with a mass deviation of less than 10 ppm from the correct molecular formula, we still encountered a combinatorial explosion in the number of candidates. This issue persisted despite applying three filtering modes in SIRIUS (STRICT, COMMON, RDBE), with the STRICT and COMMON modes even eliminating the correct molecular formula.

Experimental setting:

Strictness of the chemical filter levels are STRICT > COMMON > RDBE > NONE

Compound: PFDoA
Formula: C12HF23O2
Precursor m/z: 612.9551
adduct: [M-H]-
SMILES:
O=C(O)C(F)(F)C(F)(F)C(F)(F)C(F)(F)C(F)(F)C(F)(F)C(F)(F)C(F)(F)C(F)(F)C(F)(F)C(F)(F)F
Example of a shell command used in SIRIUS:
sirius decomp --mass "612.9551" --elements "C[0-]N[0-]O[0-]H[0-]S[0-5]P[0-3]I[0-1]Cl[0-1]Br[0-1]F[0-25]" --filter "COMMON" --ppm 10 --ion "[M-H]-" --output "test_file.csv"

Number of formula candidates (STRICT mode): 18786
Correct formula in candidates (STRICT mode): No
Number of formula candidates (COMMON mode): 20798
Correct formula in candidates (COMMON mode): No
Number of formula candidates (RDBE mode): 34101
Correct formula in candidates (RDBE mode): Yes
Number of formula candidates (NONE mode): 90378
Correct formula in candidates (NONE mode): Yes

S3 Parameter Settings for MS data processing

An in-house R script was developed to execute this analysis. Raw mass spectrometry data were imported using the ‘readMSData’ function from the MSnbase package (version 2.34.0) in R. Parameter optimization for the CentWave algorithm in XCMS was performed using the IPO package[1]. Specifically, the ‘optimizeXcmsSet’ function from the IPO package was used to determine optimal parameters for 'min_peakwidth', 'max_peakwidth', 'ppm', 'mzdiff', 'snthresh'. Peak detection in wastewater samples was subsequently performed using the ‘findChromPeaks’ function from XCMS with the optimized parameters. Detected chromatographic peaks were exported as comma-separated values (CSV) files. Corresponding MS/MS spectra (MS Level 2) for the detected peaks were extracted using the ‘chromPeakSpectra’ function from the Spectra (version 1.16.0) and MsBackendMsp packages (version 1.10.0)[2], all MS/MS data were exported in MSP format.

S4 HRMS Data from Standards Dataset std_150

The authentic reference standards for the 25 target PFAS analytes and mixtures of isotopically labeled compounds used as internal standards (ISs) were obtained from Wellington Laboratories (Guelph, Ontario, Canada). In addition, eleven authentic standards were procured to confirm PFAS identities through non-targeted analysis. For further details, refer to the *Materials* section in the doctoral dissertation of Chen[3] (https://tdr.lib.ntu.edu.tw/retrieve/e6a0d00b-326e-41e4-bc87-1567f316c5e5/ntu-112-2.pdf). Notably, the standard dataset (std_150) comprised only 27 PFAS compounds, corresponding to 176 MS^2 spectra. One compound, FBSEE diol, was excluded due to its detection as an acetate adduct ($[M+CH_3COO]^-$), which was not compatible with the study's precursor ion selection criteria.

Table S1: Chemicals list of the training set of the std_150 dataset. It includes 13 unique PFAS compounds with chemical name, molecular formula, molecular weight, canonical SMILES, PubChem CID, and the number of measured spectra (a total of 55 spectra)

Name	Formula & molecular weight	Canonical SMILES	PubChem CID	Number of Spectra
PFBSi (Perfluorobutanesulfinic acid)	C4HF9O2S 284.1001	O=S(O)C(F)(F)C(F)(F)C(F)(F)C(F)(F)F	596595	7
PFDoA (Perfluoro-n-dodecanoic acid)	C12HF23O2 614.0989	O=C(O)C(F)(F)C(F)(F)C(F)(F)C(F)(F)C(F)(F)C(F)(F)C(F)(F)C(F)(F)C(F)(F)C(F)(F)C(F)(F)F	67545	4
FOSA (Perfluoro-1-octanesulfonamide)	C8H2F17NO2S 499.1449	NS(=O)(=O)C(F)(F)C(F)(F)C(F)(F)C(F)(F)C(F)(F)C(F)(F)C(F)(F)C(F)(F)F	697785	5
PFOA (Perfluoro-n-octanoic acid)	C8HF15O2 414.0687	O=C(O)C(F)(F)C(F)(F)C(F)(F)C(F)(F)C(F)(F)C(F)(F)C(F)(F)F	9554	2
PFPeA (Perfluoro-n-pentanoic acid)	C5HF9O2 264.0640	O=C(O)C(F)(F)C(F)(F)C(F)(F)C(F)(F)F	75921	3
PFBS (Perfluoro-1-butanesulfonate)	C4HF9O3S 300.0995	O=S(=O)(O)C(F)(F)C(F)(F)C(F)(F)C(F)(F)F	67815	8
PFBA (Perfluoro-n-butanoic acid)	C4HF7O2 214.0385	O=C(O)C(F)(F)C(F)(F)C(F)(F)F	9777	3
PFHpA (Perfluoro-n-heptanoic acid)	C7HF13O2 364.0611	O=C(O)C(F)(F)C(F)(F)C(F)(F)C(F)(F)C(F)(F)C(F)(F)F	67818	3
PFDS (Perfluoro-1-decanesulfonate)	C10HF21O3S 600.1448	O=S(=O)(O)C(F)(F)C(F)(F)C(F)(F)C(F)(F)C(F)(F)C(F)(F)C(F)(F)C(F)(F)C(F)(F)C(F)(F)F	67636	4
N-MeFOSAA (N-methylperfluoro-1-octanesulfonamidoacetic acid)	C11H6F17NO4S 571.2077	CN(CC(=O)O)S(=O)(=O)C(F)(F)C(F)(F)C(F)(F)C(F)(F)C(F)(F)C(F)(F)C(F)(F)C(F)(F)F	22286931	4
PFHxA (Perfluoro-n-hexanoic acid)	C6HF11O2 314.0536	O=C(O)C(F)(F)C(F)(F)C(F)(F)C(F)(F)C(F)(F)F	67542	3
PFUdA	C11HF21O2 564.0913	O=C(O)C(F)(F)C(F)(F)C(F)(F)C(F)(F)C(F)(F)C(F)(F)C(F)(F)C(F)(F)C(F)(F)C(F)(F)F	77222	4

(Perfluoro-n-undecanoic acid)				
6:2 FTSA (1H,1H,2H,2H-perfluoro-1-octanesulfonate)	C8H5F13O3S 428.1678	O=S(=O)(O)CCC(F)(F)C(F)(F)C(F)(F)C(F)(F)C(F)(F)C(F)(F)F	119688	5

Table S2: Chemicals list of the testing set of the std_150 dataset. It includes 13 unique PFAS compounds with chemical name, molecular formula, molecular weight, canonical SMILES, PubChem CID, and the number of measured spectra (total 115 spectra)

Name	Formula & molecular weight	Canonical SMILES	PubChem CID	Number of Spectra
TFMS (Trifluoromethanesulfonic acid)	CHF3O3S 150.0769	O=S(=O)(O)C(F)(F)F	62406	17
PFOS (Perfluorooctanesulfonate)	C8HF17O3S 500.1297	O=S(=O)(O)C(F)(F)C(F)(F)C(F)(F)C(F)(F)C(F)(F)C(F)(F)C(F)(F)C(F)(F)F	74483	13
N-EtFOSAA (N-ethylperfluoro-1-octanesulfonamidoacetic acid)	C12H8F17NO4S 585.2343	CCN(CC(=O)O)S(=O)(=O)C(F)(F)C(F)(F)C(F)(F)C(F)(F)C(F)(F)C(F)(F)C(F)(F)C(F)(F)F	18134	3
PFPrA (Pentafluoropropionic acid)	C3HF5O2 164.0309	O=C(O)C(F)(F)C(F)(F)F	62356	9
PFHxS (Perfluorohexanesulfonic acid)	C6HF13O3S 400.1146	O=S(=O)(O)C(F)(F)C(F)(F)C(F)(F)C(F)(F)C(F)(F)C(F)(F)F	67734	12
PFDA (Perfluoro-n-decanoic acid)	C10HF19O2 514.0838	O=C(O)C(F)(F)C(F)(F)C(F)(F)C(F)(F)C(F)(F)C(F)(F)C(F)(F)C(F)(F)C(F)(F)F	9555	4
FBSA (Perfluorobutylsulfonamide)	C4H2F9NO2S 299.1147	NS(=O)(=O)C(F)(F)C(F)(F)C(F)(F)C(F)(F)F	10958205	20
PFPeS (Perfluoro-1-pentanesulfonate)	C5HF11O3S 350.1070	O=S(=O)(O)C(F)(F)C(F)(F)C(F)(F)C(F)(F)C(F)(F)F	75922	5
PFHpS (Perfluoro-1-heptanesulfonate)	C7HF15O3S 450.1221	O=S(=O)(O)C(F)(F)C(F)(F)C(F)(F)C(F)(F)C(F)(F)C(F)(F)C(F)(F)F	67820	5
PFNS (Perfluoro-1-nonanesulfonate)	C9F19O3S- 550.1372	O=S(=O)(O)C(F)(F)C(F)(F)C(F)(F)C(F)(F)C(F)(F)C(F)(F)C(F)(F)C(F)(F)C(F)(F)F	86998	5
FBSE (1,2,2,3,3,4,4,4-Nonafluoro-N-(2-hydroxyethyl)-1-butanesulfonamide)	C6H6F9NO3S 343.1674	O=S(=O)(NCCO)C(F)(F)C(F)(F)C(F)(F)C(F)(F)F	12576037	16
4:2 FTSA	C6H5F9O3S	O=S(=O)(O)CCC(F)(F)C(F)(F)C(F)(F)C(F)(F)F	20734543	3

(1H,1H,2H,2H-perfluoro-1-hexanesulfonate)	328.1527			
8:2 FTSA (1H,1H,2H,2H-perfluoro-1-decanesulfonate)	C10H5F17O3S 528.1829	O=S(=O)(O)CCC(F)(F)C(F)(F)C(F)(F)C(F)(F)C(F)(F)C(F)(F)C(F)(F)C(F)(F)F	3016044	3

S5 HRMS Data from NIST PFAS Database

Table S3: Criteria for PFAS Identification at Various Confidence Levels and Statistical Number of Spectra in NIST PFAS Database

Confidence	Import text	Number of Spectra
Confirmed by reference standard	Level 1a	10515
Indistinguishable from the reference standard	Level 1b	5133
Probably by library spectrum match	Level 2a	0
Probable by diagnostic fragmentation evidence	Level 2b	0
Probable by diagnostic homologue evidence	Level 2c	0
Positional isomer candidates	Level 3a	0
Fragmentation-based candidate	Level 3b	8743
Circumstantial candidate based on fragmentation	Level 3c	0
Circumstantial candidate based on homologues	Level 3d	0
Unequivocal molecular formula	Level 4	60
PFAS suspect screening exact mass match	Level 5a	0
Nontarget PFAS exact mass of interest	Level 5b	0

*The criteria table is based on Database Infrastructure for Mass Spectrometry - Per- and Polyfluoroalkyl Substances from NIST. See **Table 1** used in the work of Charbonnet et al[4] for details about the criteria.

Table S4: The chemical list of the training set from the NIST PFAS dataset. It includes 20 unique PFAS compounds with chemical name, molecular formula, molecular weight, canonical SMILES, PubChem CID, and the number of measured spectra (a total of 1227 spectra)

Name	Formula & molecular weight	Canonical SMILES	PubChem CID	Number of MS2 Spectra
Perfluoro(4-methoxybutanoic) acid	C5HF9O3 280.0454	O=C(O)C(F)(F)C(F)(F)C(F)(F)OC(F)(F)F	12498036	143
4,8-Dioxa-3H-perfluorononanoic acid	C7H2F12O4 378.0695	O=C(O)C(F)(F)C(F)OC(F)(F)C(F)(F)C(F)(F)OC(F)(F)F	52915299	102
N-Methylperfluorooctanesulfonamide	C9H4F17NO2S 513.1715	CNS(=O)(=O)C(F)(F)C(F)(F)C(F)(F)C(F)(F)C(F)(F)C(F)(F)C(F)(F)C(F)(F)F	3034468	46
Hexafluoroamylene glycol	C5H6F6O2 212.0905	OCC(F)(F)C(F)(F)C(F)(F)CO	67831	41
Octafluoroadipamide	C6H4F8N2O2 288.0956	N=C(O)C(F)(F)C(F)(F)C(F)(F)C(F)(F)C(=N)O	314191	24
Perfluorobutane ether sulfonate	C4HF9O4S 316.0989	O=S(=O)(O)C(F)(F)C(F)(F)OC(F)(F)C(F)(F)F	2776108	120
Perfluorononanoate	C9HF17O2 464.0762	O=C(O)C(F)(F)C(F)(F)C(F)(F)C(F)(F)C(F)(F)C(F)(F)C(F)(F)C(F)(F)F	67821	98
Perfluoro(2-((6-chlorohexyl)oxy) ethane sulfonic acid)	C8HClF16O4S 532.5836	O=S(=O)(O)C(F)(F)C(F)(F)OC(F)(F)C(F)(F)C(F)(F)C(F)(F)C(F)(F)C(F)(F)Cl	22568738	111
Perfluorohexanesulfonamide	C6H2F13NO2S 399.1298	NS(=O)(=O)C(F)(F)C(F)(F)C(F)(F)C(F)(F)C(F)(F)C(F)(F)F	11603678	182
((Perfluorooctyl)ethyl)phosphonic acid	C10H6F17O3P 528.0998	O=P(O)(O)CCC(F)(F)C(F)(F)C(F)(F)C(F)(F)C(F)(F)C(F)(F)C(F)(F)C(F)(F)F	22630493	37
Perfluorohexadecanoic acid	C16HF31O2 814.1290	O=C(O)C(F)(F)C(F)(F)C(F)(F)C(F)(F)C(F)(F)C(F)(F)C(F)(F)C(F)(F)C(F)(F)C(F)(F)C(F)(F)C(F)(F)C(F)(F)C(F)(F)C(F)(F)F	106027	31
Perfluoro-2-methyl-3-oxahexanoic acid	C6HF11O3 330.0530	O=C(O)C(F)(OC(F)(F)C(F)(F)C(F)(F)F)C(F)(F)F	114481	36

Perfluoro-3,6-dioxaheptanoic acid	C5HF9O4 296.0448	O=C(O)C(F)(F)OC(F)(F)C(F)(F)OC(F)(F)F	2782393	74
11:1 Fluorotelomer alcohol	C12H3F23O 600.1153	OCC(F)(F)C(F)(F)C(F)(F)C(F)(F)C(F)(F)C(F)(F)C(F)(F)C(F)(F)C(F)(F)C(F)(F)C(F)(F)F	2760321	12
Perfluoropentanamide	C5H3F8NO 245.0708	N=C(O)C(F)(F)C(F)(F)C(F)(F)C(F)F	2063303	37
3:3 Fluorotelomer carboxylic acid	C6H5F7O2 242.0917	O=C(O)CCC(F)(F)C(F)(F)C(F)(F)F	2774909	32
1H,1H,9H-Perfluorononyl acrylate	C12H6F16O2 486.1497	C=CC(=O)OCC(F)(F)C(F)(F)C(F)(F)C(F)(F)C(F)(F)C(F)(F)C(F)(F)C(F)F	107516	4
Octafluoroadipic acid	C6H2F8O4 290.0651	O=C(O)C(F)(F)C(F)(F)C(F)(F)C(F)(F)C(=O)O	67640	30
1H,1H,7H-Dodecafluoro-1-heptanol	C7H4F12O 332.0871	OCC(F)(F)C(F)(F)C(F)(F)C(F)(F)C(F)(F)C(F)F	67639	25
Perfluoro-4-isopropoxybutanoic acid	C7HF13O3 380.0605	O=C(O)C(F)(F)C(F)(F)C(F)(F)OC(F)(C(F)(F)F)C(F)(F)F	45075727	42

Table S5: Chemical list of the testing set from the NIST PFAS dataset. It includes 19 unique PFAS compounds with chemical name, molecular formula, molecular weight, canonical SMILES, PubChem CID, and the number of measured spectra (total 798 spectra)

Name	Formula & molecular weight	Canonical SMILES	PubChem CID	Number of MS2 Spectra
Perfluoro-3-methoxypropanoic acid	C4HF7O3 230.0379	O=C(O)C(F)(F)C(F)(F)OC(F)(F)F	120228	139
N-Ethylperfluorooctanesulfonamide	C10H6F17NO2S 527.1982	CCNS(=O)(=O)C(F)(F)C(F)(F)C(F)(F)C(F)(F)C(F)(F)C(F)(F)C(F)(F)C(F)(F)F	77797	43
Perfluorotetradecanoate	C14HF27O2 714.1139	O=C(O)C(F)(F)C(F)(F)C(F)(F)C(F)(F)C(F)(F)C(F)(F)C(F)(F)C(F)(F)C(F)(F)C(F)(F)C(F)(F)C(F)(F)C(F)(F)F	67822	66
(Heptafluorobutanoyl)pivaloylmethane	C10H11F7O2 296.1823	CC(C)(C)C(=O)CC(=O)C(F)(F)C(F)(F)C(F)(F)F	28614	76
Perfluorotridecanoate	C13HF25O2 664.1064	O=C(O)C(F)(F)C(F)(F)C(F)(F)C(F)(F)C(F)(F)C(F)(F)C(F)(F)C(F)(F)C(F)(F)C(F)(F)C(F)(F)C(F)(F)F	3018355	71
2-[(8-Chloro-1,1,2,2,3,3,4,4,5,5,6,6,7,7,8,8-hexadecafluorooctyl)oxy]-1,1,2,2-tetrafluoroethane-1-sulfonic acid	C10HClF20O4S 632.5987	O=S(=O)(O)C(F)(F)C(F)(F)OC(F)(F)C(F)(F)C(F)(F)C(F)(F)C(F)(F)C(F)(F)C(F)(F)C(F)(F)Cl	15099039	85
1-(Perfluorofluorooctyl)propane-2,3-diol	C11H7F17O2 494.1453	OCC(O)CC(F)(F)C(F)(F)C(F)(F)C(F)(F)C(F)(F)C(F)(F)C(F)(F)C(F)(F)F	2776406	13
Heptafluorobutyramide	C4H2F7NO 213.0537	N=C(O)C(F)(F)C(F)(F)C(F)(F)F	12626	24
(Perfluorobutyryl)-2-thenoylmethane	C10H5F7O2S 322.1995	O=C(CC(=O)C(F)(F)C(F)(F)C(F)(F)F)c1cccs1	68411	88
Perfluorostearic acid	C18HF35O2 914.1441	O=C(O)C(F)(F)C(F)(F)C(F)(F)C(F)(F)C(F)(F)C(F)(F)C(F)(F)C(F)(F)C(F)(F)C(F)(F)C(F)(F)C(F)(F)C(F)(F)C(F)(F)C(F)(F)C(F)(F)C(F)(F)F	167547	26

1H,1H,5H-Perfluoropentanol	C5H4F8O 232.0720	OCC(F)(F)C(F)(F)C(F)(F)C(F)F	9641	17
Perfluorooctanamide	C8H2F15NO 413.0839	N=C(O)C(F)(F)C(F)(F)C(F)(F)C(F)(F)C(F)(F)C(F)(F)C(F)(F)F	67919	5
2-(Perfluorohexyl)ethylphosphonic acid	C8H6F13O3P 428.0847	O=P(O)(O)CCC(F)(F)C(F)(F)C(F)(F)C(F)(F)C(F)(F)C(F)(F)F	53756597	51
3-Perfluoroheptylpropanoic acid	C10H5F15O2 442.1219	O=C(O)CCC(F)(F)C(F)(F)C(F)(F)C(F)(F)C(F)(F)C(F)(F)C(F)(F)F	2783376	42
6:1 Fluorotelomer alcohol	C7H3F13O 350.0776	OCC(F)(F)C(F)(F)C(F)(F)C(F)(F)C(F)(F)C(F)(F)F	550386	12
3-(Perfluoro-2-butyl)propane-1,2-diol	C7H7F9O2 294.1152	OCC(O)CC(F)(F)C(F)(F)C(F)(F)C(F)(F)F	2782554	16
1H,1H,6H,6H-Perfluorohexane-1,6-diol diacrylate	C12H10F8O4 370.1931	C=CC(=O)OCC(F)(F)C(F)(F)C(F)(F)C(F)(F)COC(=O)C=C	2775863	16
Nonafluoropentanamide	C5H2F9NO 263.0631	N=C(O)C(F)(F)C(F)(F)C(F)(F)C(F)(F)F	4191132	3
2-(Perfluorooctyl)ethanthiol	C10H5F17S 480.1847	FC(F)(F)C(F)(F)C(F)(F)C(F)(F)C(F)(F)C(F)(F)C(F)(F)C(F)(F)CCS	542755	5

Table S6: Evaluation of 19 PFAS compounds in the Test Set of NIST PFAS Database Using Metric for Accessing Substructural Similarity, Chemical Fingerprint Similarity, and Annotation Accuracy

Compound	MCS_{ratio}	MCS_{tan}	MCS_{ovrlap}	FPS_{sim}	Number of spectra with correct identification	Confidence Level	Total number of MS2 Spectra	Annotation Accuracy
Perfluoro -3-methoxypropanoic acid	Max: 0.92<br>Min: 0.63<br>Avg: 0.78	Max: 0.57<br>Min: 0.36<br>Avg: 0.46	Max: 0.92<br>Min: 0.63<br>Avg: 0.78	Max: 0.70<br>Min: 0.24<br>Avg: 0.40	8	100%	139	5.7%
N-Ethylperfluorooct anesulfonamide	Max: 0.84<br>Min: 0.67<br>Avg: 0.76	Max: 0.77<br>Min: 0.58<br>Avg: 0.68	Max: 0.90<br>Min: 0.81<br>Avg: 0.86	Max: 0.53<br>Min: 0.28<br>Avg: 0.33	0	100%	43	0%
Perfluorotetra decanoate	Max: 0.94<br>Min: 0.53<br>Avg: 0.76	Max: 0.93<br>Min: 0.44<br>Avg: 0.72	Max: 0.99<br>Min: 0.68<br>Avg: 0.92	Max: 1.00<br>Min: 0.37<br>Avg: 0.79	29	100%	66	43.9%
(Heptafluorobutanoyl) pivaloylmethane	Max: 0.79<br>Min: 0.66<br>Avg: 0.73	Max: 0.51<br>Min: 0.42<br>Avg: 0.48	Max: 0.79<br>Min: 0.66<br>Avg: 0.73	Max: 0.52<br>Min: 0.27<br>Avg: 0.42	0	100%	76	0%
Perfluorotrid ecanoate	Max: 0.95<br>Min: 0.54<br>Avg: 0.77	Max: 0.94<br>Min: 0.43<br>Avg: 0.72	Max: 0.99<br>Min: 0.43<br>Avg: 0.72	Max: 1.00<br>Min: 0.48<br>Avg: 0.81	43	100%	71	60.5%
2-[(8-Chloro-1,1,2,2,3,3,4,4,5,5,6,6,7,7,8,8-hexadecafluorooctyl) oxy]-1,1,2,2-tetrafluoroethane -1-sulfonic acid	Max: 0.81<br>Min: 0.46<br>Avg: 0.61	Max: 0.79<br>Min: 0.37<br>Avg: 0.53	Max: 0.96<br>Min: 0.64<br>Avg: 0.81	Max: 0.34<br>Min: 0.95<br>Avg: 0.58	1	100%	85	1.1%
1-(Perfluorofluorooctyl) propane-2,3-diol	Max: 0.78<br>Min: 0.37<br>Avg: 0.60	Max: 0.72<br>Min: 0.26<br>Avg: 0.50	Max: 0.90<br>Min: 0.46<br>Avg: 0.73	Max: 0.56<br>Min: 0.21<br>Avg: 0.39	0	100%	13	0%
Heptafluorobutyramide	Max: 0.82<br>Min: 0.69<br>Avg: 0.75	Max: 0.44<br>Min: 0.36<br>Avg: 0.40	Max: 0.82<br>Min: 0.69<br>Avg: 0.75	Max: 0.42<br>Min: 0.20<br>Avg: 0.26	0	100%	24	0%
(Perfluorobutyryl) -2-thenoylmethane	Max: 0.69<br>Min: 0.49<br>Avg: 0.61	Max: 0.53<br>Min: 0.29<br>Avg: 0.40	Max: 0.72<br>Min: 0.49<br>Avg: 0.61	Max: 0.37<br>Min: 0.21<br>Avg: 0.30	0	100%	88	0%
Perfluorostearic acid	Max: 0.83<br>Min: 0.47<br>Avg: 0.68	Max: 0.81<br>Min: 0.38<br>Avg: 0.65	Max: 0.98<br>Min: 0.66<br>Avg: 0.94	Max: 1.00<br>Min: 0.52<br>Avg: 0.85	0	100%	26	0%
1H,1H,5H-Perfluoropentanol	Max: 1.00<br>Min: 0.79<br>Avg: 0.96	Max: 0.67<br>Min: 0.45<br>Avg: 0.62	Max: 1.00<br>Min: 0.79<br>Avg: 0.96	Max: 0.61<br>Min: 0.25<br>Avg: 0.47	0	100%	17	0%
Perfluorooctanamide	Max: 0.98<br>Min: 0.67<br>Avg: 0.86	Max: 0.95<br>Min: 0.50<br>Avg: 0.71	Max: 0.98<br>Min: 0.68<br>Avg: 0.86	Max: 0.74<br>Min: 0.32<br>Avg: 0.45	2	100%	5	40%
2-(Perfluorohexyl) ethylphosphonic acid	Max: 0.83<br>Min: 0.44<br>Avg: 0.70	Max: 0.73<br>Min: 0.31<br>Avg: 0.57	Max: 0.86<br>Min: 0.50<br>Avg: 0.75	Max: 0.45<br>Min: 0.21<br>Avg: 0.34	0	100%	51	0%
3-Perfluoroheptyl propanoic acid	Max: 0.84<br>Min: 0.37<br>Avg: 0.61	Max: 0.76<br>Min: 0.25<br>Avg: 0.48	Max: 0.91<br>Min: 0.42<br>Avg: 0.69	Max: 0.74<br>Min: 0.20<br>Avg: 0.43	0	100%	42	0%
6:1 Fluorotelomer alcohol	Max: 1.00<br>Min: 0.75<br>Avg: 0.91	Max: 0.94<br>Min: 0.56<br>Avg: 0.76	Max: 1.00<br>Min: 0.76<br>Avg: 0.92	Max: 0.76<br>Min: 0.38<br>Avg: 0.59	0	100%	12	0%
3-(Perfluoro-2-butyl) propane-1,2-diol	Max: 0.93<br>Min: 0.68<br>Avg: 0.83	Max: 0.73<br>Min: 0.46<br>Avg: 0.59	Max: 0.93<br>Min: 0.69<br>Avg: 0.83	Max: 0.60<br>Min: 0.26<br>Avg: 0.43	0	100%	16	0%

1H,1H,6H,6H-Perfluorohexane -1,6-diol diacrylate	Max: 0.77<br>Min: 0.51<br>Avg: 0.63	Max: 0.60<br>Min: 0.35<br>Avg: 0.45	Max: 0.78<br>Min: 0.53<br>Avg: 0.65	Max: 0.54<br>Min: 0.29<br>Avg: 0.42	0	100%	16	0%
Nonafluoropentanamide	Max: 0.90<br>Min: 0.71<br>Avg: 0.82	Max: 0.63<br>Min: 0.44<br>Avg: 0.54	Max: 0.90<br>Min: 0.71<br>Avg: 0.82	Max: 0.62<br>Min: 0.26<br>Avg: 0.38	0	100%	3	0%
2-(Perfluorooctyl) ethanthiol	Max: 0.86<br>Min: 0.45<br>Avg: 0.68	Max: 0.78<br>Min: 0.33<br>Avg: 0.58	Max: 0.94<br>Min: 0.53<br>Avg: 0.79	Max: 0.53<br>Min: 0.23<br>Avg: 0.38	0	100%	5	0%

*The evaluation criteria for **Common Substructure** and **Chemical Fingerprint Similarity** are based on the average values obtained from all spectra corresponding to the same compound. **MCS**: maximal common substructure; **FPS**: fingerprint similarity; **Confidence level**: Number of spectra with all candidates classified as PFAS, divided by the total number of spectra; **Annotation Accuracy**: Number of MS2 spectra with correct identification, divided by the total number of spectra.

S6 HRMS Data from a Wastewater Sample (WWTP3)

Ten sewage (wastewater) samples (no. 184–187, 190, 191, and 194–197) from semiconductor plants and three effluent samples (WWTP3, WWTP4, WWTP5) from industrial WWTPs were collected in November 2020 and January 2021, respectively. Please refer to the “Sample Collection” section in Chen's doctoral dissertation for details (https://tdr.lib.ntu.edu.tw/retrieve/e6a0d00b-326e-41e4-bc87-1567f316c5e5/ntu-112-2.pdf).

In this study, only the WWTP3 sample was utilized for model evaluation. The analyses encompassed both targeted and non-targeted approaches. For detailed information regarding sample analysis, refer to Table 24 within the "Terminology" section, as well as the sections "Materials," "Target Analysis," and "Non-target Analysis" in Dr. Chen's doctoral dissertation. For detailed PFAS concentrations, please refer to Tables 7 and 18 and the section "Concentrations of Nontarget PFAS" in Dr. Chen's doctoral dissertation.

Table S7: List of identified PFAS compounds categorized as CL1a/CL1b based on matching with available authentic standards in wastewater samples from WWTP3. The table includes 15 unique PFAS compounds, their chemical names, molecular formulas, molecular weights, canonical SMILES representations, and the number of measured spectra (648).

Name	Formula & molecular weight	Canonical SMILES	PubChem CID	Number of Spectra
FBSEE diol (N,N-Bis(2-hydroxyethyl)perfluorobutanesulfonamide)	C8H10F9NO4S 387.2201	O=S(=O)(N(CCO)CCO)C(F)(F)C(F)(F)C(F)(F)C(F)(F)F	118689	4[a]
6:2 FTSA (1H,1H,2H,2H-perfluoro-1-octanesulfonate)	C8H5F13O3S 428.1678	O=S(=O)(O)CCC(F)(F)C(F)(F)C(F)(F)C(F)(F)C(F)(F)C(F)(F)F	119688	1
PFBS (Perfluoro-1-butanesulfonate)	C4HF9O3S 300.0995	O=S(=O)(O)C(F)(F)C(F)(F)C(F)(F)C(F)(F)F	67815	19
FBSAA (Perfluorobutane sulfonamido acetic acid)	C6H4F9NO4S 357.151	O=C(O)CNS(=O)(=O)C(F)(F)C(F)(F)C(F)(F)C(F)(F)F	10784527	466
PFHpA (Perfluoro-n-heptanoic acid)	C7HF13O2 364.0611	O=C(O)C(F)(F)C(F)(F)C(F)(F)C(F)(F)C(F)(F)C(F)(F)F	67818	2
N-MeFBSAA (Perfluorobutanesulfonylamide(N-methyl)acetate; (N-Methyl-N-[(1,1,2,2,3,3,4,4,4-nonafluorobutyl)sulfonyl] glycine))	C7H6F9NO4S 371.1775	CN(CC(=O)O)S(=O)(=O)C(F)(F)C(F)(F)C(F)(F)C(F)(F)F	22286935	17
PFHxA (Perfluoro-n-hexanoic acid)	C6HF11O2 314.0536	O=C(O)C(F)(F)C(F)(F)C(F)(F)C(F)(F)C(F)(F)F	67452	2
FBSE (1,2,2,3,3,4,4,4-Nonafluoro-N-(2-hydroxyethyl)-1-	C6H6F9NO3S 343.1674	O=S(=O)(NCCO)C(F)(F)C(F)(F)C(F)(F)C(F)(F)F	12576037	14

butanesulfonamide)				
N-MeFBSA (N-(Methyl) nonafluorobutane sulfonamide)	C5H4F9NO2S 313.1414	CNS(=O)(=O)C(F)(F)C(F)(F)C(F)(F)C(F)(F)F	109968	4
FBSA (Perfluorobutylsulfonamide)	C4H2F9NO2S 299.1147	NS(=O)(=O)C(F)(F)C(F)(F)C(F)(F)C(F)(F)F	10958205	83
PFPeA (Perfluoro-n-pentanoic acid)	C5HF9O2 264.0460	O=C(O)C(F)(F)C(F)(F)C(F)(F)C(F)(F)F	75921	2
PFBSi (Perfluorobutanesulfinic acid)	C4HF9O2S 284.1001	O=S(O)C(F)(F)C(F)(F)C(F)(F)C(F)(F)F	596595	22
PFBA (Perfluoro-n-butanoic acid)	C4HF7O2 214.0385	O=C(O)C(F)(F)C(F)(F)C(F)(F)F	9777	2
PFPrA (Pentafluoropropionic acid)	C3HF5O2 164.0309	O=C(O)C(F)(F)C(F)(F)F	62356	4
TFMS (Trifluoromethanesulfonic acid)	CHF3O3S 150.0769	O=S(=O)(O)C(F)(F)F	62406	6

[a] The precursor ion of the measured spectra was an acetate adduct $[M+CH3COO]^-$

Table S8: Evaluation of 15 identified PFAS compounds categorized as CL1a/CL1b based on matching with available authentic standards in wastewater samples from WWTP3. The evaluation was performed using metrics for assessing substructural similarity, chemical fingerprint similarity, and annotation accuracy. Compounds such as 6:2 FTSA, PFHpA, PFHxA, PFBS, PFPeA, PFBSi, and PFBA had more than 50% of their spectra correctly identified within the top 20 candidate molecules. 86.7% of the compounds showed annotation confidence greater than 90%, while 93.3% exhibited confidence exceeding 50%.

**Compound**	MCS_{ratio}	MCS_{tan}	MCS_{ovrlap}	FPS_{sim}	**Number of spectra with correct identification**	**Confidence Level**	**Total numbe r of MS2 Spectra**	**Annotation Accuracy**
6:2 FTSA (Sodium 1H,1H,2H,2H-perfluoro-1- octanesulfonate)	Max: 1.00 Min: 0.84 Avg: 0.94	Max: 1.00 Min: 0.75 Avg: 0.79	Max: 1.00 Min: 0.88 Avg: 0.94	Max: 1.00 Min: 0.58 Avg: 0.81	1	100%	1	100%
PFHpA (Perfluoro-n-heptanoic acid)	Max: 1.00 Min: 0.73 Avg: 0.90	Max: 1.00 Min: 0.50 Avg: 0.78	Max: 1.0 Min: 0.73 Avg: 0.90	Max: 1.00 Min: 0.31 Avg: 0.71	2	100%	2	100%
FBSEE diol (N,N-Bis(2-hydroxyethyl) perfluorobutanesulfonamide)	Max: 0.67 Min: 0.45 Avg: 0.59	Max: 0.57 Min: 0.30 Avg: 0.43	Max: 0.80 Min: 0.48 Avg: 0.62	Max: 0.46 Min: 0.24 Avg: 0.36	0	100%	4	0%
FBSAA (Perfluorobutane sulfonamido acetic acid)	Max: 0.77 Min: 0.56 Avg: 0.68	Max: 0.63 Min: 0.37 Avg: 0.49	Max: 0.83 Min: 0.57 Avg: 0.69	Max: 0.63 Min: 0.27 Avg: 0.43	0	99%	466	0%
N-MeFBSAA (Perfluorobutanesulfonylamide(N-methyl)acetate; (N-Methyl-N-[(1,1,2,2,3,3,4,4,4-nonafluorobutyl)sulfonyl] glycine))	Max: 0.71 Min: 0.51 Avg: 0.63	Max: 0.62 Min: 0.36 Avg: 0.47	Max: 0.85 Min: 0.54 Avg: 0.66	Max: 0.51 Min: 0.24 Avg: 0.37	0	94%	17	0%
PFHxA (Perfluoro-n-hexanoic acid)	Max: 0.71 Min: 0.53 Avg: 0.64	Max: 0.60 Min: 0.32 Avg: 0.44	Max: 0.71 Min: 0.53 Avg: 0.64	Max: 0.59 Min: 0.29 Avg: 0.48	1	50%	2	50%
FBSE (1,2,2,3,3,4,4,4-Nonafluoro-N-(2-hydroxyethyl)-1-butanesulfonamide)	Max: 0.82 Min: 0.42 Avg: 0.65	Max: 0.77 Min: 0.28 Avg: 0.49	Max: 0.91 Min: 0.44 Avg: 0.68	Max: 0.76 Min: 0.22 Avg: 0.42	0	95%	14	0%
N-MeFBSA (N-(Methyl) nonafluorobutanesulfonamide)	Max: 0.85 Min: 0.65 Avg: 0.75	Max: 0.67 Min: 0.46 Avg: 0.52	Max: 0.87 Min: 0.66 Avg: 0.75	Max: 0.22 Min: 0.52 Avg: 0.35	0	100%	3	0%
FBSA (Perfluorobutylsulfonamide)	Max: 0.93 Min: 0.66 Avg: 0.83	Max: 0.73 Min: 0.40 Avg: 0.56	Max: 0.93 Min: 0.66 Avg: 0.83	Max: 0.82 Min: 0.26 Avg: 0.49	22	99%	83	26.5%
PFBS (Perfluoro-1-butanesulfonate)	Max: 0.97 Min: 0.70 Avg: 0.86	Max: 0.96 Min: 0.57 Avg: 0.71	Max: 0.97 Min: 0.78 Avg: 0.86	Max: 0.96 Min: 0.30 Avg: 0.66	18	95%	19	95%
PFPeA (Perfluoro-n-pentanoic acid)	Max: 0.97 Min: 0.75 Avg: 0.90	Max: 0.83 Min: 0.46 Avg: 0.64	Max: 0.97 Min: 0.75 Avg: 0.90	Max: 0.95 Min: 0.27 Avg: 0.61	1	100%	2	50%

PFBSi (Perfluorobutanesulfinic acid)	Max: 0.94<br>Min: 0.61<br>Avg: 0.82	Max: 0.88<br>Min: 0.43<br>Avg: 0.64	Max: 0.94<br>Min: 0.62<br>Avg: 0.82	Max: 0.86<br>Min: 0.23<br>Avg: 0.55	14	92%	22	63.6%
PFBA (Perfluoro-n-butanoic acid)	Max: 1.00<br>Min: 0.77<br>Avg: 0.90	Max: 1.00<br>Min: 0.59<br>Avg: 0.71	Max: 1.00<br>Min: 0.77<br>Avg: 0.90	Max: 1.00<br>Min: 0.19<br>Avg: 0.56	2	100%	2	100%
PFPrA (Pentafluoropropionic acid)	Max: 0.60<br>Min: 0.17<br>Avg: 0.41	Max: 0.34<br>Min: 0.09<br>Avg: 0.21	Max: 0.60<br>Min: 0.17<br>Avg: 0.41	Max: 0.15<br>Min: 0.04<br>Avg: 0.08	0	25%	4	0%
TFMS (Trifluoromethanesulfonic acid)	Max: 0.94<br>Min: 0.50<br>Avg: 0.74	Max: 0.46<br>Min: 0.20<br>Avg: 0.33	Max: 0.94<br>Min: 0.50<br>Avg: 0.74	Max: 0.19<br>Min: 0.04<br>Avg: 0.10	0	100%	6	0%

*The evaluation criteria for Common Substructure and Chemical Fingerprint Similarity are based on the average values obtained from all spectra for each compound. MCS: maximal common substructure; FPS: fingerprint similarity; Confidence Level: Number of spectra with all candidates classified as PFAS, divided by the total number of spectra; Annotation Accuracy: Number of MS2 spectra with correct identification, divided by the total number of spectra.

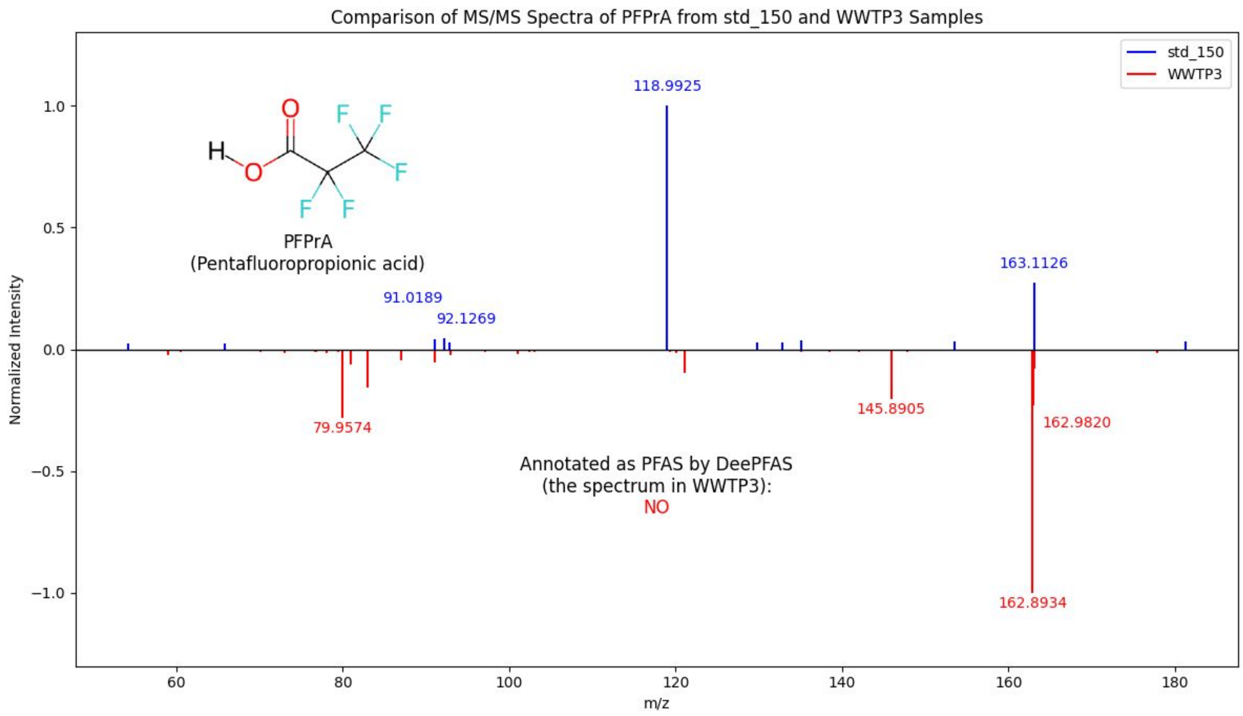

Figure S1: Comparison of MS/MS Spectra of PFPrA from std_150 and WWTP3 Samples. Peak intensities were normalized to the maximum intensity within each spectrum. The four most intense peaks were annotated with their corresponding m/z values.

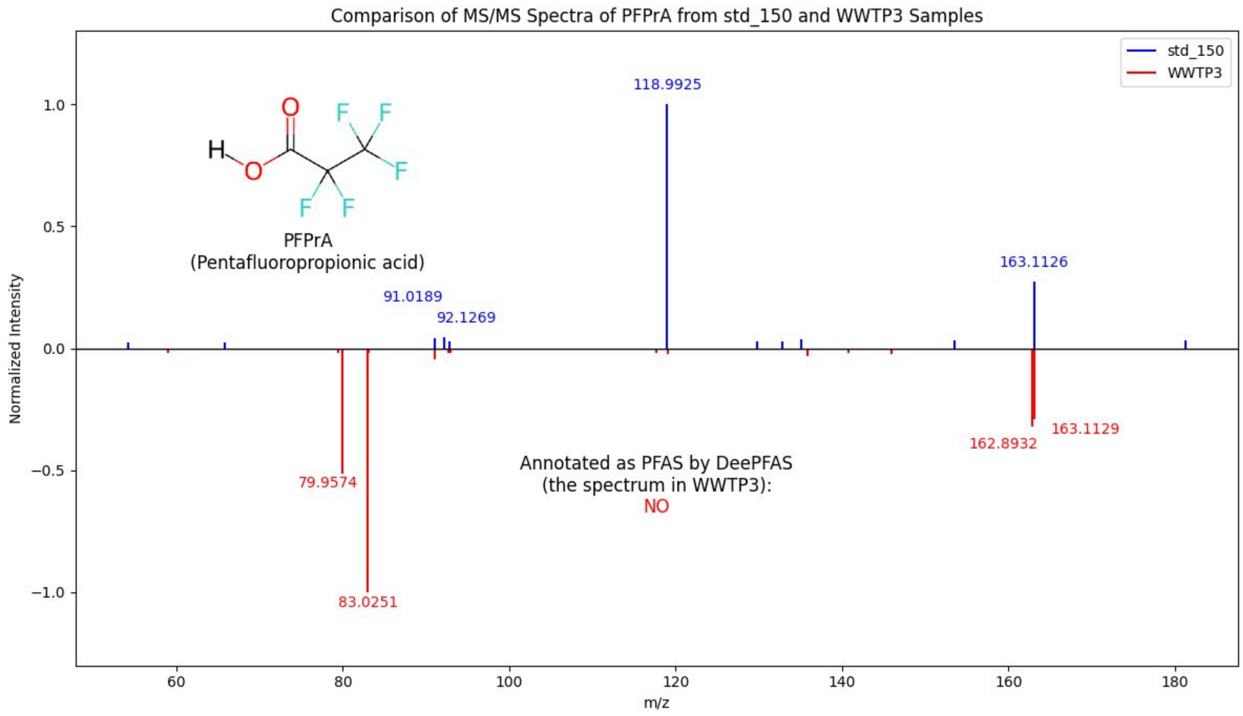

Figure S2: Comparison of MS/MS Spectra of PFPrA from std_150 and WWTP3 Samples. Peak intensities were normalized to the maximum intensity within each spectrum. Additionally, These peaks were annotated with their corresponding m/z values.

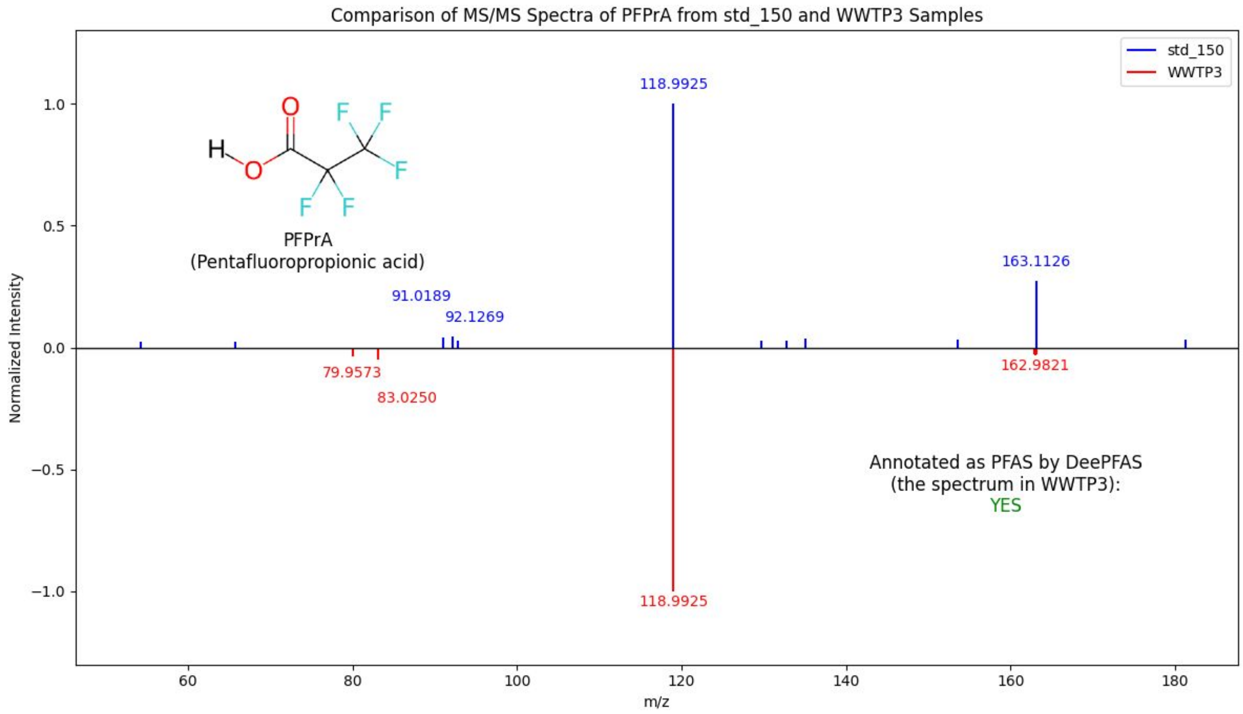

Figure S3: Comparison of MS/MS Spectra of PFPrA from std_150 and WWTP3 Samples. Peak intensities were normalized to the maximum intensity within each spectrum. The four most intense peaks were annotated with their corresponding m/z values.

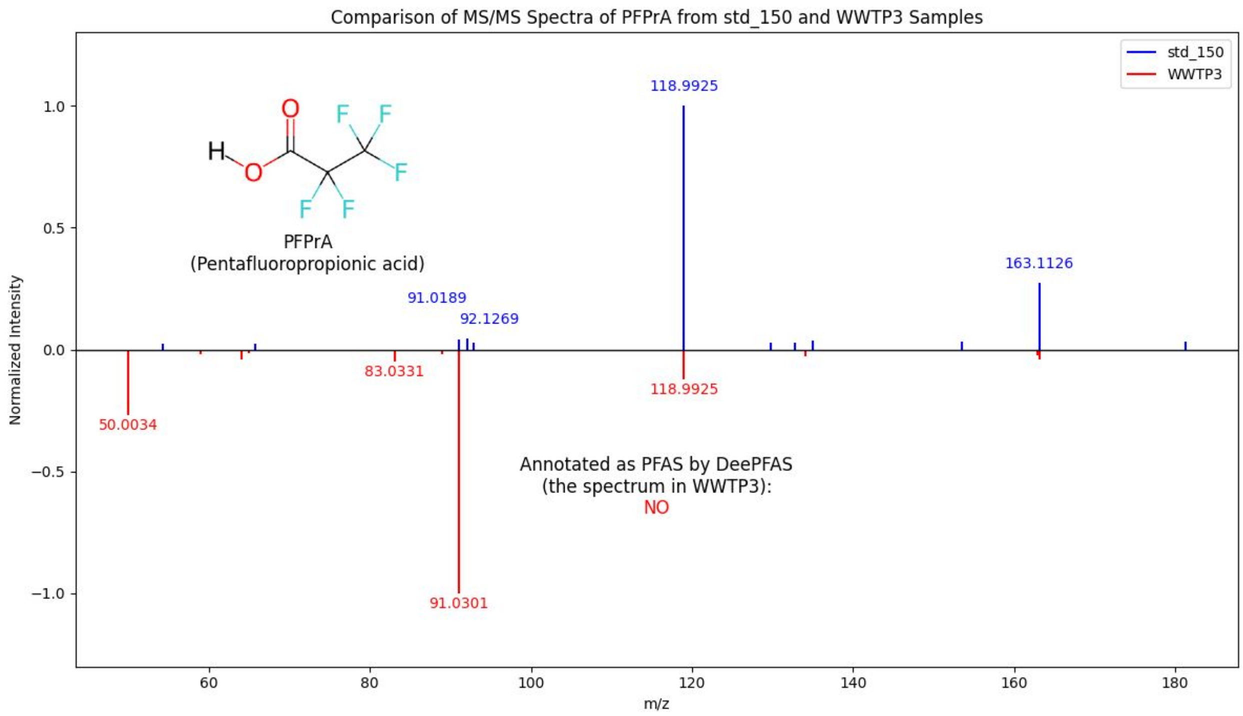

Figure S4: Comparison of MS/MS Spectra of PFPrA from std_150 and WWTP3 Samples. Peak intensities were normalized to the maximum intensity within each spectrum. Additionally, these peaks were annotated with their corresponding m/z values.

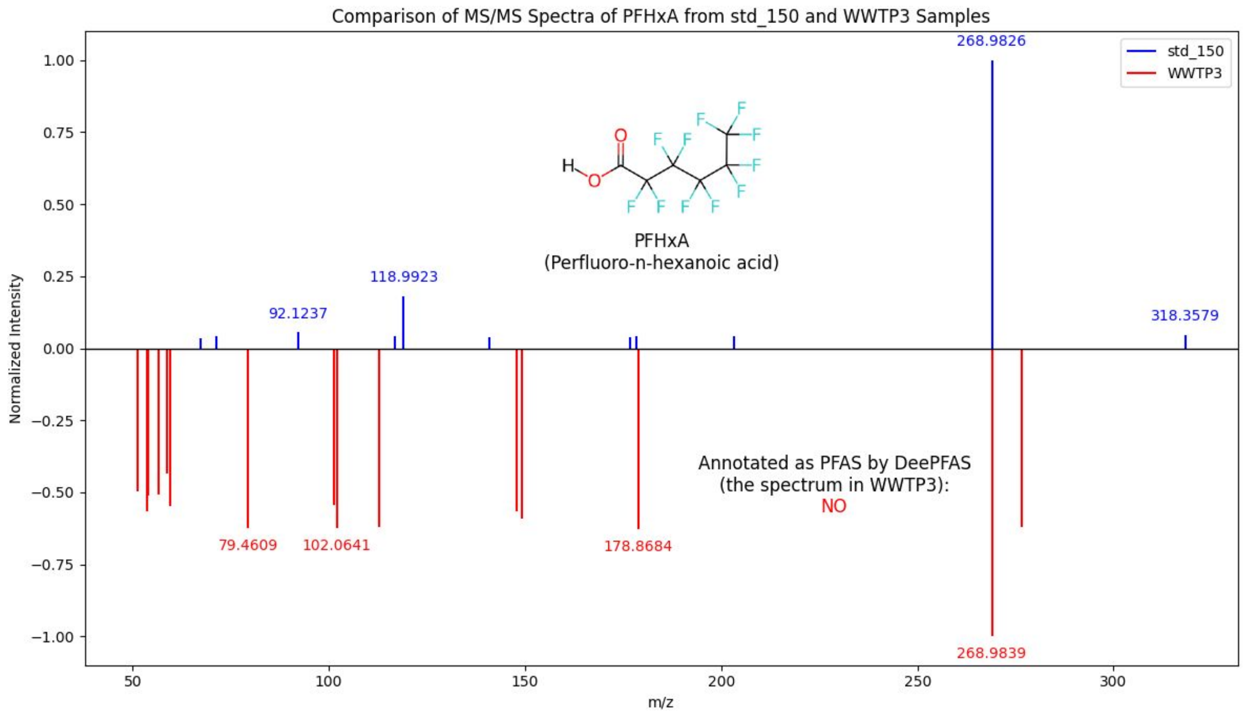

Figure S5: Comparison of MS/MS Spectra of PFHxA from std_150 and WWTP3 Samples. Peak intensities were normalized to the maximum intensity within each spectrum. The four most intense peaks were annotated with their corresponding m/z values.

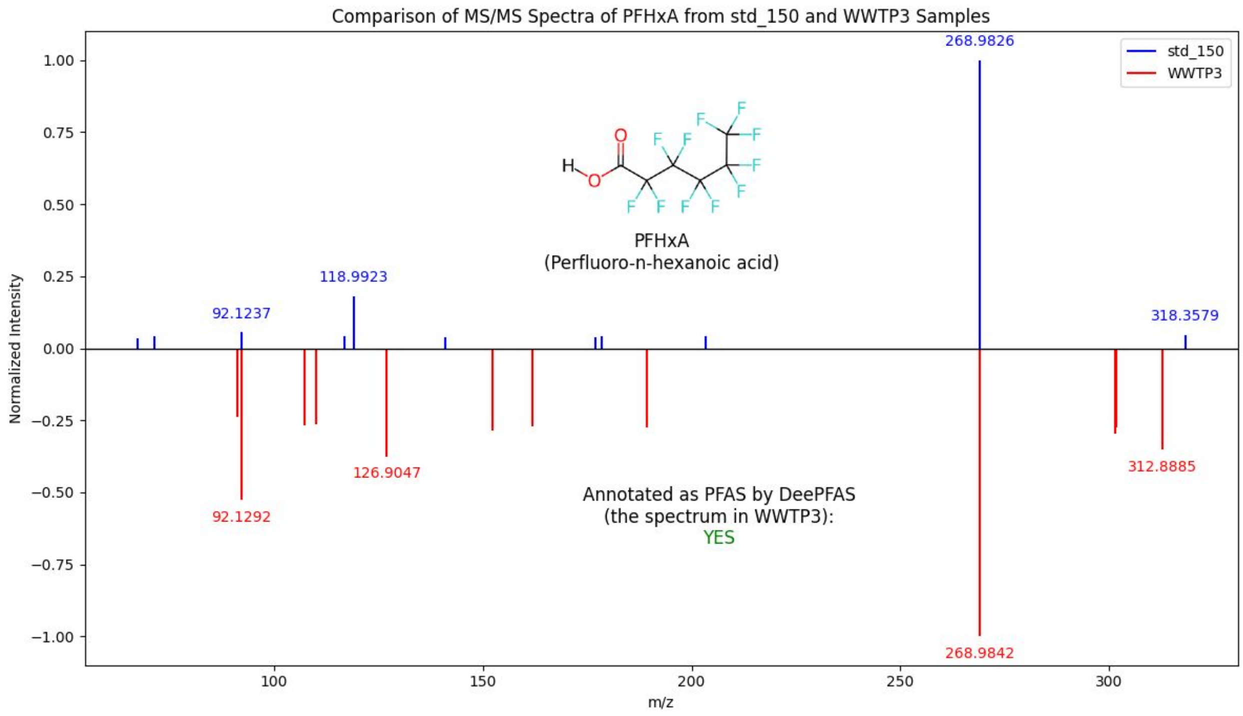

Figure S6: Comparison of MS/MS Spectra of PFHxA from std_150 and WWTP3 Samples. Peak intensities were normalized to the maximum intensity within each spectrum. The four most intense peaks were annotated with their corresponding m/z values.

Table S9: List of identified PFAS compounds categorized as CL2b to CL3c in wastewater samples from WWTP3. This table includes 24 unique PFAS compounds, their chemical names, molecular formulas, molecular weights, canonical SMILES representations, and the number of measured spectra (a total of 86 spectra). For detailed information, please refer to Table 24 and the appendix section in the doctoral dissertation of Dr. Chen (https://tdr.lib.ntu.edu.tw/retrieve/e6a0d00b-326e-41e4-bc87-1567f316c5e5/ntu-112-2.pdf).

Name	Formula & molecular weight	Canonical SMILES	PubChem CID	Number of Spectra
FBSEE mono-ol monoacid (N-(2-hydroxyethyl) perfluoroalkane sulfonamido acetic acids)	C8H8F9NO5S 401.2036	O=C(O)CN(CCO)S(=O)(=O)C(F)(F)C(F)(F) C(F)(F)C(F)(F)F	NA	4
FBSEE diacid (2,2'-(((Nonafluorobut yl)sulfonyl)imino)diacetic acid)	C8H6F9NO6S 415.1871	O=C(O)CN(CC(=O)O)S(=O)(=O)C(F)(F)C(F)(F)C(F)(F)C(F)(F)F	1653634 38	19
PFdiCA (C6) (Octafluoroadipic acid)	C6H2F8O4 290.0652	O=C(O)C(F)(F)C(F)(F)C(F)(F)C(F)(F)C(=O) O	67640	1
H-PFHxA (2,2,3,3,4,4,5,5,6, 6-decafluorohexano ic acid)	C6H2F10O2 296.0632	O=C(O)C(F)(F)C(F)(F)C(F)(F)C(F)(F)C(F)F	1492299 7	3
H-FBSA (Hydro-substituted perfluoroalkyl sulfonamides)	C4H3F8NO2S 281.1243	NS(=O)(=O)C(F)(F)C(F)(F)C(F)(F)C(F)F	1653655 65	3
PFdiCA (C5) (Perfluoroglutaric acid)	C5H2F6O4 240.0576	O=C(O)C(F)(F)C(F)(F)C(F)(F)C(=O)O	67827	2
E-PFBA (difluoro(pentaflu oroethoxy)acetic acid)	C4HF7O3 230.038	O=C(O)C(F)(F)OC(F)(F)C(F)(F)F	1249803 2	2
H-PFPrS (1,1,2,2,3,3-hexafluoropropan e-1-sulfonic acid)	C3HF5O3 232.1015	O=S(=O)(O)C(F)(F)C(F)(F)C(F)F	1840278 3	1
PFdiCA (C4) (Perfluorosuccini c acid)	C4H2F4O4 190.0500	O=C(O)C(F)(F)C(F)(F)C(=O)O	67833	3
H-PFBA (2,2,3,3,4,4-hexafluorobutanoi c acid)	C4H2F6O2 196.0481	O=C(O)C(F)(F)C(F)(F)C(F)F	1752534	2
E-PFPrA (difluoro(trifluoro	C3HF5O3 180.0304	O=C(O)C(F)(F)OC(F)(F)F	5125273	1

methoxy)acetic acid)				
H_2-E-PFPrS (1,2,2-trifluoro-2-(1,2,2,2-tetrafluoroethoxy) ethane-1-sulfonic acid)	C4H3F7O4S 280.1180	O=S(=O)(O)C(F)C(F)(F)OC(F)C(F)(F)F	NA	3
U-E-PFPeA (Difluoro[(1,1,2,3,3-pentafluoroprop-2-en-1-yl)oxy]acetic acid)<br><br>U-E-PFPeA (difluoro[[(1E)-1,2,3,3,3-pentafluoroprop-1-en-1-yl]oxy]acetic acid)	C5HF7O3 242.0487	O=C(O)C(F)(F)OC(F)(F)C(F)=C(F)F<br><br>O=C(O)C(F)(F)OC(F)=C(F)C(F)(F)F	NA<br><br>139595311	1
U-E-PFHxA (Difluoro{[(2E)-1,1,2,3,4,4,4-heptafluorobut-2-en-1-yl]oxy}acetic acid)<br><br>U-E-PFHxA ((3E)-2,2,3,4-tetrafluoro-4-(pentafluoroethoxy)but-3-enoic acid)<br><br>U-E-PFHxA (2,2,3,3,4,4-hexafluoro-4-[(trifluoroethenyl)oxy]butanoic acid)	C6HF9O3 292.0562	O=C(O)C(F)(F)OC(F)(F)C(F)=C(F)C(F)(F)F<br><br>O=C(O)C(F)(F)C(F)=C(F)OC(F)(F)C(F)(F)F<br><br>O=C(O)C(F)(F)C(F)(F)C(F)(F)OC(F)=C(F)F	NA<br><br>NA<br><br>NA	4
H-FBSAA ([(1,1,2,2,3,3,4,4-	C6H5F8NO4S 339.1605	O=C(O)CNS(=O)(=O)C(F)(F)C(F)(F)C(F)(F)C(F)F	NA	3

octafluorobutane-1-sulfonyl)amino]acetic acid)				
FBSA-Am (N-carbamoyl-1,1,2,2,3,3,4,4,4-nonafluorobutane-1-sulfonamide)	C5H3F9N2O3S 342.1396	NC(=O)NS(=O)(=O)C(F)(F)C(F)(F)C(F)(F)C(F)(F)F	NA	2
FBSA-MeAm (2-(1,1,2,2,3,3,4,4,4-nonafluorobutane-1-sulfonamido)acetamide)	C6H5F9N2O3S 356.1662	NC(=O)CNS(=O)(=O)C(F)(F)C(F)(F)C(F)(F)C(F)(F)F	NA	3
FBSAcAL (enol form) (1,1,2,2,3,3,4,4,4-nonafluoro-N-[(E)-2-hydroxyethenyl]butane-1-sulfonamide)<br><br>FBSAcAL (keto form) (1,1,2,2,3,3,4,4,4-nonafluoro-N-(2-oxoethyl)butane-1-sulfonamide)	C6H4F9NO3S 341.1516	O=S(=O)(NC=CO)C(F)(F)C(F)(F)C(F)(F)C(F)(F)F<br><br>O=CCNS(=O)(=O)C(F)(F)C(F)(F)C(F)(F)C(F)(F)F	NA<br><br>101393213	5
FBSE-SO_3H ((2-hydroxyethyl)(1,1,2,2,3,3,4,4,4-nonafluorobutane-1-sulfonyl)sulfamic acid)	C6H6F9NO6S2 423.2304	O=S(=O)(O)N(CCO)S(=O)(=O)C(F)(F)C(F)(F)C(F)(F)C(F)(F)F	NA	4
E-FBSAA ([1,1,2,2,3,3-hexafluoro-3-(trifluoromethoxy)propane-1-sulfonamido]acetic acid)	C6H4F9NO5S 373.1504	O=C(O)CNS(=O)(=O)C(F)(F)C(F)(F)C(F)(F)OC(F)(F)F<br><br>O=C(O)CNS(=O)(=O)C(F)(F)C(F)(F)OC(F)(F)C(F)(F)F	NA<br><br>NA	10

E-FBSAA ([1,1,2,2-tetrafluoro-2-(pentafluoroethoxy)ethanesulfonamido]acetic acid)				
E-FBSA (1,1,2,2,3,3-hexafluoro-3-(trifluoromethoxy)propane-1-sulfonamide)<br><br>E-FBSA (1,1,2,2-tetrafluoro-2-(pentafluoroethoxy)ethane-1-sulfonamide)	C4H2F9NO3S 315.1142	NS(=O)(=O)C(F)(F)C(F)(F)C(F)(F)OC(F)(F)F<br><br>NS(=O)(=O)C(F)(F)C(F)(F)OC(F)(F)C(F)(F)F	NA<br><br>NA	1
FBSE-Am (N-carbamoyl-1,1,2,2,3,3,4,4,4-nonafluoro-N-(2-hydroxyethyl)butane-1-sulfonamide)	C7H7F9N2O4S 386.1922	NC(=O)N(CCO)S(=O)(=O)C(F)(F)C(F)(F)C(F)(F)C(F)(F)F	NA	2
FBSA-SO_3H ((1,1,2,2,3,3,4,4,4-nonafluorobutane-1-sulfonyl)sulfamic acid)	C4H2F9NO5S2 379.1778	O=S(=O)(O)NS(=O)(=O)C(F)(F)C(F)(F)C(F)(F)C(F)(F)F	NA	2
FBSAcAL hemiacetal (1,1,2,2,3,3,4,4,4-nonafluoro-N-(2-hydroxy-2-methoxyethyl)butane- 1-sulfonamide)	C6H6F9NO4S 359.1668	O=S(=O)(NCC(O)O)C(F)(F)C(F)(F)C(F)(F)C(F)(F)F	NA	5

Table S10: Evaluation of 24 identified PFAS compounds categorized as CL2b to CL3c in wastewater samples from WWTP3. The evaluation was performed using metrics for assessing substructural similarity, chemical fingerprint similarity, and annotation accuracy. 79.1% of the compounds had 100% annotation confidence, while 95.8% exhibited confidence exceeding 50%. 13 PFAS compounds were identified by PFΔScreen.

Compound	MCS_{ratio}	MCS_{tan}	MCS_{ovrlap}	FPS_{sim}	Confidence Level	Total number of MS2 Spectra	Annotation Accuracy	PFΔScreen
FBSEE mono-ol monoacid (N-(2-hydroxyethyl) perfluoroalkane sulfonamido acetic acids)	Max: 0.69<br>Min: 0.51<br>Avg: 0.60	Max: 0.63<br>Min: 0.37<br>Avg: 0.47	Max: 0.88<br>Min: 0.58<br>Avg: 0.69	Max: 0.41<br>Min: 0.29<br>Avg: 0.42	100%	4	0%	True
FBSEE diacid (2,2'-(((Nonafluorobutyl) sulfonyl)imino)diacetic acid)	Max: 0.61<br>Min: 0.48<br>Avg: 0.54	Max: 0.49<br>Min: 0.33<br>Avg: 0.39	Max: 0.72<br>Min: 0.52<br>Avg: 0.59	Max: 0.23<br>Min: 0.45<br>Avg: 0.34	100%	19	0%	True
PFdiCA (C6) (Octafluoroadipic acid)	Max: 0.89<br>Min: 0.67<br>Avg: 0.76	Max: 0.76<br>Min: 0.52<br>Avg: 0.66	Max: 0.89<br>Min: 0.71<br>Avg: 0.83	Max: 0.93<br>Min: 0.22<br>Avg: 0.58	100%	1	0%	False
H-PFHxA (2,2,3,3,4,4,5,5,6,6-decafluorohexanoic acid)	Max: 0.78<br>Min: 0.48<br>Avg: 0.56	Max: 0.70<br>Min: 0.32<br>Avg: 0.53	Max: 0.78<br>Min: 0.48<br>Avg: 0.65	Max: 0.22<br>Min: 0.48<br>Avg: 0.68	100%	3	0%	True
H-FBSA (Hydro-substituted perfluoroalkyl sulfonamides)	Max: 1.00<br>Min: 0.71<br>Avg: 0.92	Max: 0.70<br>Min: 0.41<br>Avg: 0.61	Max: 1.00<br>Min: 0.71<br>Avg: 0.92	Max: 0.86<br>Min: 0.29<br>Avg: 0.52	100%	3	0%	False
PFdiCA (C5) (Perfluoroglutaric acid)	Max: 0.87<br>Min: 0.40<br>Avg: 0.58	Max: 0.73<br>Min: 0.27<br>Avg: 0.37	Max: 0.87<br>Min: 0.41<br>Avg: 0.58	Max: 0.54<br>Min: 0.11<br>Avg: 0.19	100%	2	0%	True
E-PFBA (Difluoro(pentafluoroethoxy)acetic acid)	Max: 0.43<br>Min: 0.18<br>Avg: 0.32	Max: 0.21<br>Min: 0.98<br>Avg: 0.14	Max: 0.43<br>Min: 0.18<br>Avg: 0.32	Max: 0.14<br>Min: 0.10<br>Avg: 0.11	50%	2	0%	True
H-PFPrS (1,1,2,2,3,3-hexafluoropropane-1-sulfonic acid)	Max: 1.00<br>Min: 0.69<br>Avg: 0.77	Max: 0.76<br>Min: 0.45<br>Avg: 0.48	Max: 1.00<br>Min: 0.69<br>Avg: 0.77	Max: 0.66<br>Min: 0.15<br>Avg: 0.27	100%	1	0%	False
PFdiCA (C4) (Perfluorosuccinic acid)	Max: 0.89<br>Min: 0.58<br>Avg: 0.78	Max: 0.62<br>Min: 0.33<br>Avg: 0.47	Max: 0.89<br>Min: 0.58<br>Avg: 0.62	Max: 0.53<br>Min: 0.15<br>Avg: 0.33	100%	3	0%	False
H-PFBA (2,2,3,3,4,4-hexafluorobutanoic acid)	Max: 0.88<br>Min: 0.38<br>Avg: 0.55	Max: 0.58<br>Min: 0.18<br>Avg: 0.30	Max: 0.88<br>Min: 0.38<br>Avg: 0.55	Max: 0.44<br>Min: 0.09<br>Avg: 0.18	50%	2	0%	False
E-PFPrA (Difluoro(trifluoromethoxy)acetic acid)	Max: 0.45<br>Min: 0.36<br>Avg: 0.45	Max: 0.22<br>Min: 0.15<br>Avg: 0.20	Max: 0.45<br>Min: 0.36<br>Avg: 0.45	Max: 0.10<br>Min: 0.07<br>Avg: 0.08	100%	1	0%	False
FBSA-SO_3H ((1,1,2,2,3,3,4,4,4-nonafluorobutane-1-sulfonyl)sulfamic acid)	Max: 1.00<br>Min: 0.62<br>Avg: 0.76	Max: 0.81<br>Min: 0.44<br>Avg: 0.58	Max: 0.81<br>Min: 0.57<br>Avg: 0.72	Max: 0.70<br>Min: 0.32<br>Avg: 0.50	100%	2	0%	True
H_2-E-PFPrS	NA	NA	NA	NA	33.3%	3	NA	True

(1,2,2-trifluoro-2-(1,2,2,2-tetrafluoroethoxy)ethane-1-sulfonic acid)								
U-E-PFPeA (isomers)	NA	NA	NA	NA	100%	1	NA	False
U-E-PFHxA (isomers)	NA	NA	NA	NA	100%	4	NA	True
H-FBSAA ([(1,1,2,2,3,3,4,4-octafluorobutane-1-sulfonyl)amino]acetic acid)	Max: 0.90<br>Min: 0.69<br>Avg: 0.76	Max: 0.72<br>Min: 0.44<br>Avg: 0.57	Max: 0.82<br>Min: 0.60<br>Avg: 0.73	Max: 0.74<br>Min: 0.28<br>Avg: 0.47	100%	3	0%	True
FBSA-Am (N-carbamoyl-1,1,2,2,3,3,4,4,4-nonafluorobutane-1-sulfonamide)	Max: 0.88<br>Min: 0.57<br>Avg: 0.73	Max: 0.75<br>Min: 0.37<br>Avg: 0.52	Max: 0.88<br>Min: 0.57<br>Avg: 0.73	Max: 0.75<br>Min: 0.31<br>Avg: 0.48	100%	2	0%	True
FBSA-MeAm (2-(1,1,2,2,3,3,4,4,4-nonafluorobutane-1-sulfonamido)acetamide)	Max: 0.56<br>Min: 0.28<br>Avg: 0.38	Max: 0.37<br>Min: 0.15<br>Avg: 0.24	Max: 0.49<br>Min: 0.25<br>Avg: 0.38	Max: 0.42<br>Min: 0.20<br>Avg: 0.29	66.7%	3	0%	True
FBSAcAL (isomers)	NA	NA	NA	NA	100%	5	NA	False
FBSE-SO_3H ((2-hydroxyethyl) (1,1,2,2,3,3,4,4,4-nonafluorobutane-1-sulfonyl)sulfamic acid)	Max: 0.64<br>Min: 0.32<br>Avg: 0.44	Max: 0.45<br>Min: 0.16<br>Avg: 0.27	Max: 0.59<br>Min: 0.24<br>Avg: 0.38	Max: 0.43<br>Min: 0.16<br>Avg: 0.30	75%	4	0%	False
E-FBSAA (isomers)	NA	NA	NA	NA	100%	10	NA	True
E-FBSA (isomers)	NA	NA	NA	NA	100%	1	NA	True
FBSE-Am (N-carbamoyl-1,1,2,2,3,3,4,4,4-nonafluoro-N-(2-hydroxyethyl)butane-1-sulfonamide)	Max: 0.57<br>Min: 0.33<br>Avg: 0.42	Max: 0.38<br>Min: 0.19<br>Avg: 0.27	Max: 0.50<br>Min: 0.30<br>Avg: 0.41	Max: 0.36<br>Min: 0.25<br>Avg: 0.32	100%	2	0%	False
FBSAcAL hemiacetal (1,1,2,2,3,3,4,4,4-nonafluoro-N-(2-hydroxy-2-methoxyethyl)butane- 1-sulfonamide)	Max: 0.99<br>Min: 0.59<br>Avg: 0.79	Max: 0.81<br>Min: 0.37<br>Avg: 0.58	Max: 0.82<br>Min: 0.50<br>Avg: 0.68	Max: 0.76<br>Min: 0.22<br>Avg: 0.45	100%	5	0%	False

*The evaluation criteria for Common Substructure and Chemical Fingerprint Similarity are based on the average values obtained from all spectra for each compound. MCS: maximal common substructure; FPS: fingerprint similarity; Confidence Level: Number of spectra with all candidates classified as PFAS, divided by the total number of spectra; Annotation Accuracy: Number of MS2 spectra with correct identification, divided by the total number of spectra; PFΔScreen: Indicates whether the compound was annotated as PFAS by PFΔScreen. A value of "NA" indicates the presence of isomers of the corresponding identified PFAS in the row. Consequently, the correct molecule for the isomers corresponding to the spectra cannot be determined due to the lack of annotated information.

S7 Parameter Settings for PFΔScreen in the Comparative Analysis with DeePFAS

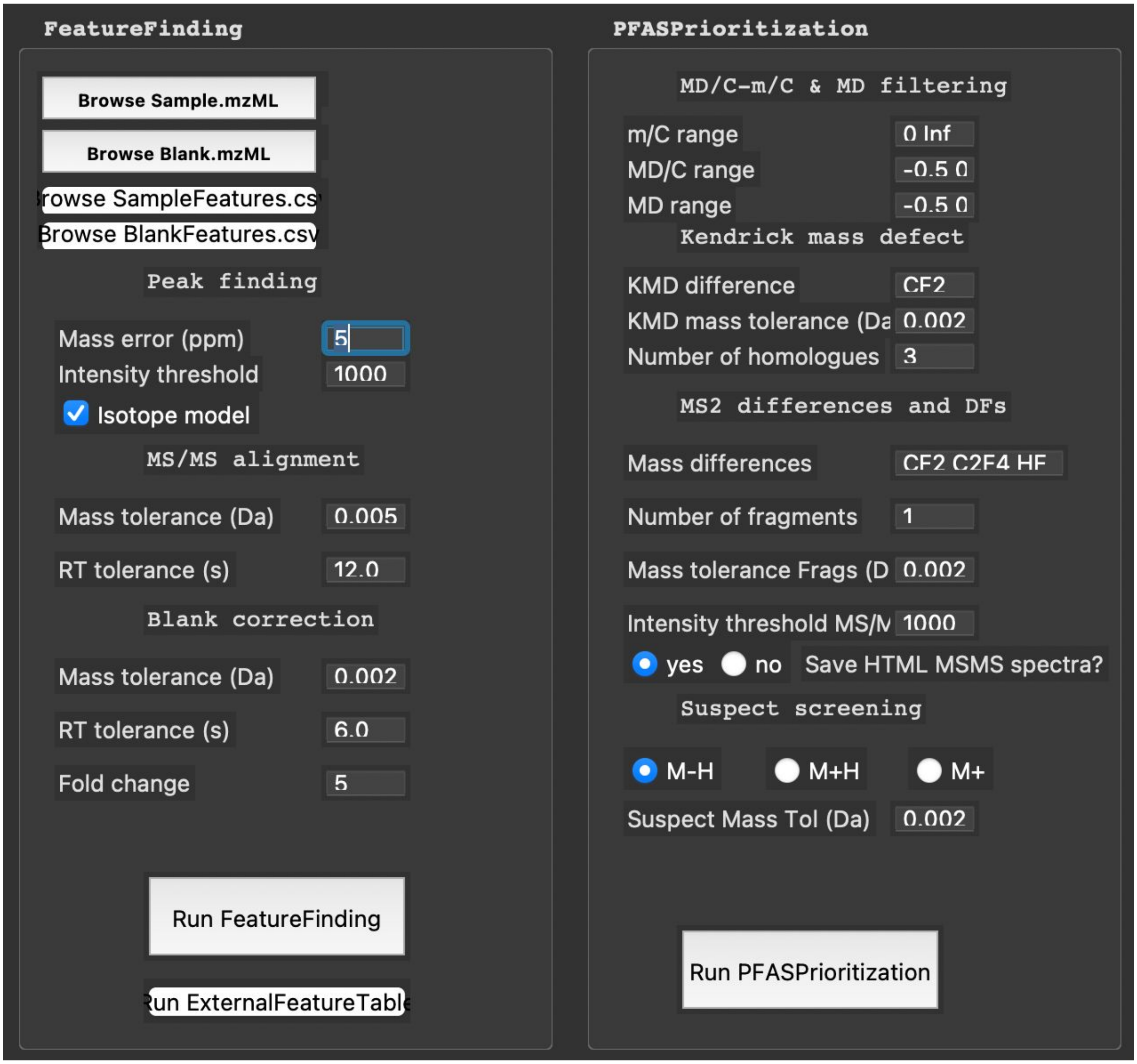

Figure S7: The Parameter Settings for PFΔScreen in the Comparative Analysis with DeePFAS.

S8 Model Architecture and Optimization

During the autoencoder training, we incorporated additional task-specific constraints to make the latent space generated by the encoder more chemically meaningful. Specifically, in addition to translating randomized SMILES to canonical SMILES, the latent space was also required to predict various chemical properties of the molecules. These properties include molecular formula, logP, molar refractivity, number of valence electrons, number of hydrogen bond donors and acceptors, Balaban's J value, topological polar surface area, drug likeness (QED), and synthetic accessibility (SA). We used two fully connected layers with dimensions of 512 and 128, respectively, along with a Rectified Linear Unit (ReLU) activation function, to construct a compact neural network for learning these chemical features. These chemical features were also calculated using the RDKit toolkit (version 2023.9.5).

Both the autoencoder and spectra encoder were optimized using the Adam optimizer with eps 5e-9. The DeePFAS model was developed and trained using PyTorch[5], a deep learning framework implemented in Python (version 3.1.0).

Table S11 Model hyperparameters searched for the autoencoder and spectra encoder

Model	Parameter	Grid	Value
	learning rate		1e-4
	batch size	{1e-3, 1e-4, 1e-5}	1024
	dropout	{128, 256, 512, 1024, 2048}	0.1
	head	{0, 0.1, 0.2, 0.3, 0.4, 0.5, 0.6}	8
	embedding size	{4, 8, 12}	512
	hidden size	{256, 512}	2048
	layers	{1024, 2048}	3
	spectrum resolution	{3, 4}	0.01
	oversampling	{0.1, 0.01}	True
		{True, False}	
**Spectra Encoder**			
	CNN kernel sets (first dimension of 2D-CNN)	{{3, 4, 5}, {2, 3, 4, 5}, {2, 3, 4, 5, 10}, {2, 3, 4, … , 10}, {2, 3, 4, … , 10, 20, 50}, {2, 3, 4, … 19, 20, 50}, {3, 4, 5, 10 ,20, 50}}	{2, 3, 4, …, 10, 20, 50}
	Number of filters in each kernel	{100, 200, 256, 300, 512, 1024}	256
		{max, avg}	
	CNN pooling		max
	learning rate	{1e-5, 1e-4, 3e-4, 1e-3}	3e-4
	batch size	{128, 256, 512}	256
	embedding size	{256, 512}	512
**AutoEncoder**	hidden size	{256, 512}	512
	bidirectional	{True, False}	True
	layers of the encoder	{2, 3, 4}	3
	layers of decoder	{2, 3, 4}	3

S9 Data Availability

We used the NIST2020 LC-ESI-MS/MS library, which is commercial and can be purchased from NIST's licensed distributor. In DeePFAS, the HRAM MS/MS spectra and associated compounds used were exported to MSP/MOL format by the free software Lib2NIST Library Conversion Tool offered by NIST (https://chemdata.nist.gov/mass-spc/ms-search/Library_conversion_tool.html). The PFAS standards from the std_150 dataset and the wastewater sample (WWTP3) were provided by the National Environmental Research Academy, Ministry of Environment, Taiwan. These datasets are also publicly available for download at https://zenodo.org/records/15770612 and https://zenodo.org/records/15354850, respectively. The NIST PFAS Database (version 1.1) is a public resource and can be downloaded in SQLite format from https://data.nist.gov/od/id/mds2-2905, accessed on January 2, 2025.

The molecular dataset used in this study consists of approximately 200 million molecules collected from the PubChem and ZINC-20 databases. PubChem and ZINC-20 are publicly available datasets containing around 100 million and 1 billion molecules, respectively. These datasets were downloaded on April 10, 2024, and August 17, 2024, from https://ftp.ncbi.nlm.nih.gov/pubchem/Compound/Extras/ and https://files.docking.org/zinc20-ML/, respectively.

S10 Code Availability

Our work is publicly available at https://github.com/CMDM-Lab/DeePFAS. The GitHub repository contains the source code for data processing and model implementation, details on Python package versions, and the trained model weights.

S11 Supplementary References

(1) Libiseller, G.; Dvorzak, M.; Kleb, U.; Gander, E.; Eisenberg, T.; Madeo, F.; Neumann, S.; Trausinger, G.; Sinner, F.; Pieber, T.; Magnes, C. IPO: A Tool for Automated Optimization of XCMS Parameters. *BMC Bioinformatics* **2015**, *16* (1), 118. https://doi.org/10.1186/s12859-015-0562-8.

(2) *A Modular and Expandable Ecosystem for Metabolomics Data Annotation in R*. https://www.mdpi.com/2218-1989/12/2/173 (accessed 2024-11-20).

(3) Chen, Y.-J. Detection and Discovery of Novel Perfluoroalkyl and Polyfluoroalkyl Substances (PFAS) in the Environment – A Case Study of Semiconductor Wastewater, 2024. https://tdr.lib.ntu.edu.tw/handle/123456789/93571?mode=full.

(4) Charbonnet, J. A.; McDonough, C. A.; Xiao, F.; Schwichtenberg, T.; Cao, D.; Kaserzon, S.; Thomas, K. V.; Dewapriya, P.; Place, B. J.; Schymanski, E. L.; Field, J. A.; Helbling, D. E.; Higgins, C. P. Communicating Confidence of Per- and Polyfluoroalkyl Substance Identification via High-Resolution Mass Spectrometry. *Environ. Sci. Technol. Lett.* **2022**, *9* (6), 473–481. https://doi.org/10.1021/acs.estlett.2c00206.

(5) Paszke, A.; Gross, S.; Massa, F.; Lerer, A.; Bradbury, J.; Chanan, G.; Killeen, T.; Lin, Z.; Gimelshein, N.; Antiga, L.; Desmaison, A.; Köpf, A.; Yang, E.; DeVito, Z.; Raison, M.; Tejani, A.; Chilamkurthy, S.; Steiner, B.; Fang, L.; Bai, J.; Chintala, S. PyTorch: An Imperative Style, High-Performance Deep Learning Library. arXiv December 3, 2019. https://doi.org/10.48550/arXiv.1912.01703. (accessed 2024-11-27).
